# Validating HIV Viral Suppression Threshold Adjustments for Comparable Estimates Using Data From Nationally Representative Household Surveys in Sub-Saharan Africa

**DOI:** 10.1097/QAI.0000000000003878

**Published:** 2026-04-15

**Authors:** Olanrewaju Edun, Lucy Okell, Timothy M. Wolock, Eline L. Korenromp, Leigh F. Johnson, Jeffrey W. Imai-Eaton

**Affiliations:** a MRC Centre for Global Infectious Disease Analysis, School of Public Health, Imperial College London, London, United Kingdom;; b Data for Impact Department, Joint United Nations Programme on HIV/AIDS, Geneva, Switzerland;; c Centre for Integrated Data and Epidemiological Research, University of Cape Town, Cape Town, South Africa; and; d Center for Communicable Disease Dynamics, Department of Epidemiology, Harvard T.H. Chan School of Public Health, Boston, MA

**Keywords:** viral load suppression, antiretroviral therapy, Population-based HIV Impact Assessment survey, distributional regression, viral load distribution

## Abstract

**Introduction:**

To enable comparable global assessments of viral load suppression (VLS) among people living with HIV on antiretroviral therapy, the Joint United Nations Programme on HIV/AIDS applies a model to adjust VLS estimates reported at different thresholds to a common viral load ≤1000 copies/mL definition. We assessed performance of the current reverse Weibull model and alternatives using survey data from sub-Saharan Africa.

**Methods:**

Using data from 21 Population-based HIV Impact Assessment surveys in 16 sub-Saharan African countries (2015–2022), we assessed 6 models (Weibull, reverse Weibull, Pareto, Fréchet, gamma, and lognormal) in adjusting VLS reported at viral load <50, <200, <400 copies/mL to ≤1000. We compared predictions using parameters from Johnson et al and recalibrated using Population-based HIV Impact Assessment surveys, assessing whether new shape parameters improved adjustments and varied by sex and age.

**Results:**

In adjustments from all thresholds, the Weibull model had the lowest prediction errors (root-mean-squared error for <200 to ≤1000: Weibull: 1.9%; reverse Weibull: 3.1%; Pareto: 2.5%). Prediction errors for reverse Weibull and Pareto models were higher in subgroups with low VLS compared with Weibull. Across 21 surveys, in adjustments from <200 to ≤1000, reverse Weibull overestimated VLS by 2.3%, compared with 1.5% by Weibull and Pareto. The Fréchet, gamma, and lognormal models performed similarly to Weibull. Shape parameter estimates for the Weibull and reverse Weibull were slightly higher after recalibration and varied by sex and age.

**Conclusions:**

The Weibull, Fréchet, gamma, and lognormal models provided more reliable VLS adjustments across thresholds than the previously recommended reverse Weibull model, avoiding inflated VLS estimates that could obscure gaps in HIV treatment programs and underestimate HIV transmission risks.

## Abstract 2

Supplemental Digital Content is Available in the Text.

## INTRODUCTION

Monitoring HIV viral load (VL) among people living with HIV (PLHIV) is important for assessing antiretroviral therapy (ART) effectiveness and HIV transmission risk.^1^ The Joint United Nations Programme on HIV/AIDS (UNAIDS) “95-95-95” targets aim for 95% of PLHIV on ART to achieve viral load suppression (VLS) by 2025 (“third 95” target), defined as VL ≤1000 copies/mL.^2,3^ However, some national programs use different thresholds (<400, <200, or <50 copies/mL) in national monitoring,^4–7^ leading to spurious differences in VLS across settings or even within a single setting if not accounted for.^8–11^

To standardize global monitoring of progress toward the “third 95” target, UNAIDS apply a mathematical formula to adjust VLS estimates from country-reported thresholds to the common VL ≤1000 threshold.^8^ The adjustment is based on analysis by Johnson et al^8^ that fitted Weibull, Pareto, and reverse Weibull models to VL distribution data from PLHIV on ART in the International epidemiology Databases to Evaluate AIDS (IeDEA) collaboration and the ART Cohort Collaboration (ART-CC). The IeDEA and ART-CC data included PLHIV from 7 global regions, including sub-Saharan Africa, and Europe from 2010 to 2019. The calibrated models were validated by assessing their reliability in adjusting VLS estimates using World Health Organization (WHO) drug resistance report data (from Guatemala, Honduras, Nicaragua, Vietnam, and Zambia) and Brazil's national ART program. Although Pareto and reverse Weibull models best fit the calibration data (assessed using the log likelihood statistics), the Weibull model performed best in validation with the WHO data,^8^ although all 3 model predictions fell within uncertainty limits of the validation data. The Pareto model performed best on validation with the Brazilian program data, but its uncertainty ranges were the narrowest. Given the Weibull's relatively poorer goodness-of-fit to calibration data and the Pareto's narrow uncertainty ranges, the study recommended the reverse Weibull model for adjusting estimates of VLS, which UNAIDS incorporated into the Spectrum model in 2021. For example, an observed 80% VLS at a threshold of <200 copies/mL from routine VL monitoring would be adjusted to 88.3% at ≤1000 copies/mL using the reverse Weibull model.

Because of the limited availability of individual-level VL data, the models were calibrated to aggregate VL data, which may have limited their ability to assess individual-level variability and extreme values, influencing shape parameter estimates. Data were limited to evaluate differences in the VL distributions among individuals by age and sex.^8^ In addition, because of limited data, the performance of the models in adjusting VLS estimates were validated mostly using data from South and central America, with limited validation against data from sub-Saharan Africa, where rates of HIV drug resistance may be lower.^12^

Using nationally representative Population-based HIV Impact Assessment surveys (PHIAs)^13^ from sub-Saharan Africa, we aimed to (1) evaluate the models estimated by Johnson et al in African populations; (2) compare alternative statistical models for describing observed VL distributions; and (3) assess sex and age differences in VL distribution parameters among PLHIV on ART in sub-Saharan Africa.

## METHODS

### Adjustment Approaches

Johnson et al^8^ explored 3 models—Weibull, reverse Weibull, and Pareto—to represent the cumulative distribution of VL on the log_10_ scale (log_10_-VL) among PLHIV on ART. Table 1 summarizes the cumulative distribution functions (CDFs) for each model. The reverse Weibull model imposed an upper limit of 6 log_10_-VL (1,000,000 copies/mL) on VLs among PLHIV on ART, modelling the difference between an individual's log_10_-VL and this upper limit using the Weibull CDF. Johnson et al estimated shape parameters from empirical CDFs (reported in Table 1). Then for a fixed-shape parameter, a VLS observation at threshold *t*_1_ (eg, <50, <200, <400) was converted to threshold *t*_2_ (≤1000 copies/mL) by calculating the scale parameter (formula in Table 1). All 3 models were calibrated using data from the IeDEA and ART-CC cohorts to obtain shape estimates and validated using data form the WHO drug resistance report and the Brazilian national ART program.

**TABLE 1.** Models of Viral Load Distributions Among PLHIV on ART (Adapted From Johnson et al)

Model	Cumulative Distribution Function: Probability of Viral Load Below Threshold *t*_1_*F*(*t*_1_)	Shape or SD Parameter	Scale Parameter (or Mean) as Function of Known Shape Parameter (or SD) and Quantile *F*(*t*_1_)	Probability of Viral Load Below Threshold *t*_2_ , *F*(*t*_2_), as Function of Known *F*(*t*_1_) and Shape (or SD) Parameter	Lower Limit	Upper Limit	Shape Parameter Estimates and 95% Confidence Interval (Estimated by Johnson et al.^8^)
Weibull	$1-\exp\left(-\lambda \log_{10}{\left(t_{1}\right)}^{\phi }\right)$	Φ	$\lambda =\frac{-\ln\left(1-F\left(t_{1}\right)\right)}{\log_{10}{\left(t_{1}\right)}^{\phi }}$	$1-\left(1-F\left(t_{1}\right)\right){\left(\frac{\log_{10}t_{2}}{\log_{10}t_{1}}\right)}^{\phi }$	1	∞	0.85 (0.43–1.26) (8)
Reverse Weibull	$\exp\left(-\lambda {\left(6-\log_{10}\left(t_{1}\right)\right)}^{\phi }\right)$	Φ	$\lambda =\frac{-\ln\left(F\left(t_{1}\right)\right)}{{\left(6-\log_{10}t_{1}\right)}^{\phi }}$	$F\left(t_{1}\right){\left(\frac{6-\log_{10}t_{2}}{6-\log_{10}t_{1}}\right)}^{\phi }$	0	1,000,000	2.81 (1.70–3.92) (8)
Pareto	$1-{\left(\frac{m}{\log_{10}\left(t_{1}\right)}\right)}^{\alpha }$	α	$m=\log_{10}{\left(t_{1}\right)\left(1-F\left(t_{1}\right)\right)}^{\frac{1}{\alpha }}$	$1-\left(1-F\left(t_{1}\right)\right){\left(\frac{\log_{10}\left(t_{1}\right)}{\log_{10}\left(t_{2}\right)}\right)}^{\alpha }$	$10^{m}$	∞	1.73 (1.20–2.26) (8)
Fréchet	$\exp{-\left(\frac{\log_{10}\left(t_{1}\right)}{\beta }\right)}^{-\Phi }$	Φ	$\beta =\log_{10}\left(t_{1}\right)\left({-\ln\left(F\left(t_{1}\right)\right)}^{\frac{1}{\Phi }}\right)$	$F\left(t_{1}\right){\left(\frac{\log_{10}\left(t_{2}\right)}{\log_{10}\left(t_{1}\right)}\right)}^{\phi }$	0	∞	—
Gamma	$\frac{1}{\Gamma \left(\alpha \right)}\gamma \left(\alpha ,\frac{\log_{10}\left(t_{1}\right)}{\beta }\right)$	α	Derived by solving the cumulative distribution function (CDF) for a known shape (or SD) and $F\left(t_{1}\right)$	Estimated using the derived scale (or mean) and shape (or SD) from calibration	0	∞	—
Lognormal	$\frac{1}{2}\left[1+\mathrm{erf}\left(\frac{\ln\log_{10}\left(t_{1}\right)-\mu }{\sigma \sqrt{2}}\right)\right]$	σ			0	∞	—

For the gamma distribution: β represents the scale and α the shape parameter, whereas Γ(α) is the gamma function and $\gamma \left(\alpha ,\frac{\log_{10}\left(t_{1}\right)}{\beta }\right)$ is the incomplete gamma function. For the Fréchet distribution: β represents the scale parameter and Φ is the shape parameter. For the lognormal distribution, $\mathrm{erf}\left(\frac{\ln\log_{10}\left(t_{1}\right)-\mu }{\sigma \sqrt{2}}\right)$ represents the cumulative distribution function of the lognormal distribution at $t_{1}$, where μ and σ are the mean and SD, respectively, of the underlying standard normal probability density. For all models, the shape parameter (and SD for lognormal) controls the variance of the distribution of viral loads. The scale parameter (λ for the Weibull and reverse Weibull models, m for the Pareto model, β for the gamma and Fréchet) and μ for the lognormal distribution determine the mean of the distribution.

### Data Source

We analyzed participant-level VL data from 21 PHIAs conducted in 16 sub-Saharan African countries between 2015 and 2022. These cross-sectional household surveys collected standardized, nationally representative HIV-related data.^13^ Surveys included 2 each from Eswatini, Malawi, Lesotho, Zambia, and Zimbabwe, and 1 survey from Cameroon, Côte d’Ivoire, Nigeria, Ethiopia, Kenya, Rwanda, Uganda, Tanzania, Namibia, Mozambique, and Botswana.

Details of the PHIA design, sampling, and procedures have been described previously.^14,15^ Consenting participants completed structured questionnaires including demographic information and self-reported ART use. Whole-blood samples were collected by venous draw for household-based and laboratory testing. HIV status was determined using point-of-care serological testing, with positive samples confirmed by the Geenius HIV-1/2 Confirmatory Assay (Bio-Rad, Hercules, CA), followed by HIV RNA VL testing and antiretroviral screening to assess ART use.^14,15^

Ethical approvals were obtained from institutional review boards in each country, the US Centers for Disease Control and Prevention, and Columbia University or the University of Maryland at Baltimore (Nigeria, Botswana, and Zambia 2020 surveys). Written informed consent was obtained from adult participants, with assent and parental consent required for minors (typically 15–17 years). This secondary analysis received ethical approval from the Imperial College Research Governance and Integrity Team (ICREC #20IC6451).

Our analyses included participants aged 15 years and older, HIV seropositive, and on ART based on either presence of detectable antiretrovirals in blood or self-reported ART usage.

### Statistical Analyses

First, to evaluate the models derived by Johnson et al, we analyzed the observed survey-weighted proportions of PLHIV on ART with VL <50, <200, and <400 copies/mL in each survey. These values were then input into the cumulative distributions for the Pareto, Weibull, and reverse Weibull models (Table 1) to predict the proportion with VL ≤1000 copies/mL. We compared these with the observed survey proportion with VL ≤1000, quantified prediction errors using the root-mean-squared error (RMSE), and reported the average difference between the predicted and observed proportions as a measure of bias. Lower RMSE indicated smaller prediction errors.

Second, we refit the Weibull and reverse Weibull models to individual-level log_10_-VL data from the 21 PHIAs, assuming a common shape parameter for all surveys while allowing country- and survey-specific random effects in the scale parameter. VL measurements reported at the lower limit of quantification (LLOQ) varied across and within surveys and were treated in the likelihood as censored observations at the reported LLOQ (eg, <20 or <839 copies/mL). Measurements reported as “Target not detected”, without a specified LLOQ, were censored at the lowest quantifiable VL within the survey (ranging from 20 to 40 copies/mL) (see Table S1, Supplemental Digital Content, http://links.lww.com/QAI/C685).^16–18^ To facilitate fitting the reverse Weibull model, which imposes an upper limit of 6 log_10_-VL, we truncated VL values ≥6 log_10_-VL at 5.99 log_10_-VL for 52 eligible participants with recorded VL above this threshold. We assessed model fits by comparing empirical VL histograms (log_10_ transformed) with probability density estimates of the Weibull, reverse Weibull and Pareto distributions, using both parameters from Johnson et al^8^ and those estimated from PHIA data. We additionally compared the empirical CDFs and probability density functions of the survey data with those of the fitted distributions. We assessed whether the recalibrated models improved VLS predictions at ≤1000 copies/mL using the reported proportions <50, <200, and <400 copies/mL by comparing RMSE and average bias.

Because the Pareto distribution's scale parameter *m* defines its lower limit, it was unsuitable for likelihood-based fitting to individual-level survey observations. Instead, we tested a range of plausible shape parameter values for the Pareto distribution, visually assessed their fits to PHIA data, and evaluated their predictive performance for VLS ≤ 1000 copies/mL (see Figures S2–S4, Supplemental Digital Content, http://links.lww.com/QAI/C685).

Third, we explored 3 additional distributions used for modelling right-skewed data, Fréchet, gamma, and lognormal.^19–22^ Their CDFs are in Table 1. These models were fitted to individual-level log_10_-VL data from the 21 PHIAs as described previously to obtain a common shape parameter. The fixed shape parameter and a VLS observation at threshold *t*_1_ were then used to convert to a threshold *t*_2_ (≤1000 copies/mL) by calculating the scale parameter, as described previously (Table 1). We then compared their performance against the models by Johnson et al using the RMSE and average bias.

Finally, to assess if best fitting shape parameters differed by age and sex, we fit the models to PHIA data using distributional regression, in which the shape or SD parameters vary with respect to covariates.^23^ We applied distributional regression, specifying the shape parameter as a log-linear function of sex and age categories (15–24, 25–34, 35–44, 45–54, and 55+ years), including country- and survey-specific random effects for the scale parameter. We then compared VLS predictions using these sex- and age-specific parameters to those from sex- and age-invariant parameters, using RMSE and average bias.^8^

Adjustments from <200 to ≤1000 copies/mL were used to illustrate results because most countries reporting VLS estimates to UNAIDS at alternative thresholds use <200 copies/mL (see Table S2, Supplemental Digital Content, http://links.lww.com/QAI/C685). Analyses were conducted in R version 4.3.1, with model calibration using the *brms* package (version 2.20.4).

### Sensitivity Analyses

To assess the impact of restricting analyses to individuals established on treatment (given WHO/UNAIDS recommendations to report viral suppression among those on ART for at least 6 months), we refit the models using VL data from respondents who had been on ART for ≥12 months—the shortest duration reported in the PHIA surveys. These data were available for 14 surveys conducted over 2015–2019. To determine whether any differences in parameter estimates were driven by excluding individuals on ART for <12 months, we also refit the model using all PLHIV on ART in the same 14 pre-2020 surveys.

## RESULTS

Across 21 surveys, VL measurements were available for 36,368 PLHIV on ART (age 15+ years; maximum age eligibility varied). The observed percentage virally suppressed (VL ≤ 1000 copies/mL) ranged from 73.7% in Côte d'Ivoire (2017–2018) to 97.9% in Botswana (2021) (see Table S1, Supplemental Digital Content, http://links.lww.com/QAI/C685).

### Evaluation of the Johnson et al Models Using PHIA Data

**FIGURE 1.**
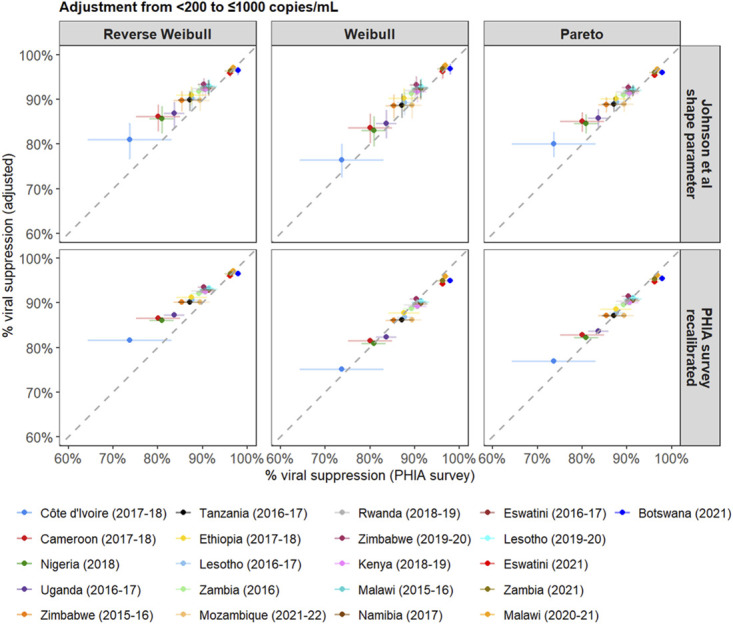
Scatter plots show the relationship between the observed percentage VLS estimates from the individual patient data in the PHIAs and the adjusted estimates (from <200 to ≤1000 copies/mL) using the reverse Weibull, Weibull and Pareto models, and parameters from Johnson et al and calibration to PHIA survey data. Shape parameter for the Pareto model on calibration to PHIA survey data used in Figure is 1.20 (see Figure S3, Supplemental Digital Content, http://links.lww.com/QAI/C685 for comparisons using other values). The surveys are arranged in ascending order based on the observed proportion of VLS at the 1000 copies/mL threshold.

Using shape parameters estimated from Johnson et al^8^ to predict VL ≤1000 copies/mL from observed proportions <50, <200, and <400 copies/mL, all 3 original models overestimated the proportion ≤1000 in most PHIAs (Fig. 1 and see Figure S1, Supplemental Digital Content, http://links.lww.com/QAI/C685). The average bias for predicted VL ≤1000 adjusting from <50, <200, and <400 for the reverse Weibull model was 1.3%, 2.3%, and 1.6%, respectively. Average bias was 0.4%, 1.5%, and 0.8% for the Weibull model and 2.0%, 1.5%, and 0.8% for the Pareto (see Table S3, Supplemental Digital Content, http://links.lww.com/QAI/C685). However, predicted VLS proportions were within survey uncertainty ranges, except for adjustments from <50 for Botswana (2021), where all 3 models significantly underestimated VLS.

The Weibull model had the smallest RMSE across adjustments: from <50 (3.0%), <200 (1.9%), and <400 (1.1%) compared with reverse Weibull (3.2%, 3.1%, 2.1%) and Pareto (3.8%, 2.5%, 1.4%). The reverse Weibull and Pareto models had larger prediction errors for surveys with lower VLS (Fig. 1 and see Figure S1, Supplemental Digital Content, http://links.lww.com/QAI/C685).

After recalibration to PHIAs, the shape parameter for the Weibull (0.91; 95% confidence interval: 0.90–0.92) and reverse Weibull (2.98; 2.93–3.02) were slightly higher but similar to Johnson et al^8^ (Weibull: 0.85; 0.43–1.26 and reverse Weibull: 2.81; 1.70–3.92). The recalibrated Weibull model improved RMSE from 1.9% to 1.4% for predictions using observed proportion <200, and from 1.1% to 0.8% for <400, but worsened from 3.0% to 4.3% for <50 (see Table S3, Supplemental Digital Content, http://links.lww.com/QAI/C685). The recalibrated reverse Weibull model slightly worsened predictions across all 3 thresholds, with larger prediction errors for surveys with lower levels of VLS. On average, it overestimated VL <1000 by 1.9%, 2.6%, and 1.8% in adjustment from <50, <200, and <400, respectively, whereas the recalibrated Weibull underestimated VLS by 3.4%, 0.8%, and 0.4%, respectively.

For Pareto, the best-fitting shape parameter varied by VLS level. Higher values (≥2.00) fit better for VLS >90%, whereas lower values (<2.00) for VLS <90% (see Figure S2, Supplemental Digital Content, http://links.lww.com/QAI/C685). Prediction errors for VL ≤1000 copies/mL were higher with shape ≥2.00 than with shape estimate (1.73) from Johnson et al, especially in lower-VLS surveys (see Figure S3, Supplemental Digital Content, http://links.lww.com/QAI/C685). A lower shape (1.20) reduced errors. RMSE for adjustment from <200 to ≤1000 was 1.4% (shape = 1.20), 2.5% (1.73), 3.1% (2.00), and 4.2% (2.50) (see Figure S3; Appendix S1; Figure S4, Supplemental Digital Content, http://links.lww.com/QAI/C685).

**FIGURE 2.**
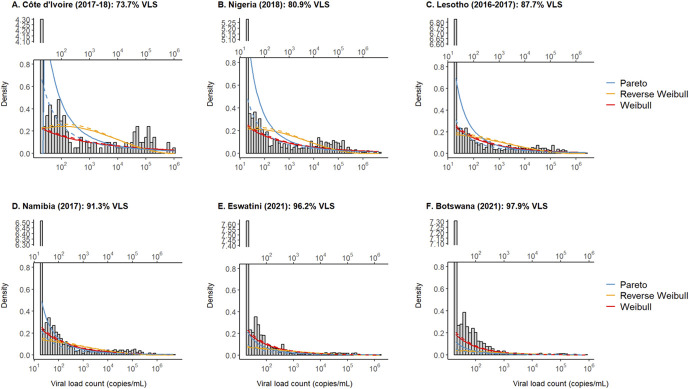
Histograms show the distribution of observed viral loads among PLHIV on ART: A, Côte d'Ivoire (2017-18); B, Côte d'Ivoire (2017-18); B, Nigeria (2018); C, Lesotho (2016–2017); D, Namibia (2018); E, Eswatini (2021); and F, Botswana (2021) PHIA surveys. Lines show the probability density estimates for the Pareto, reverse Weibull, and Weibull models using shape parameters from Johnson et al (solid line) and calibration to PHIA surveys (dashed lines). The dashed line for the Pareto model is for shape = 1.20 (see Figure S2, Supplemental Digital Content, http://links.lww.com/QAI/C685 for other values). Note: The scale parameters were set so the cumulative probability of a viral load ≤1000 copies/mL is the same as VLS estimated from the survey data. Breaks in the *y* axis were included to allow clearer visualization of the distribution (see full *y* axis in see Figure S11, Supplemental Digital Content, http://links.lww.com/QAI/C685). Gaps in the histogram for Côte d'Ivoire reflect sparse data points for certain viral load values on the *x* axis because of the few PLHIV on ART (n = 207).

Comparisons of fitted model densities and empirical VL distributions showed all models underestimated the upper tail (VL >1000 copies/mL), especially in surveys with VLS <90% (Figs. 2A–C, see Figures S9 and S10, Supplemental Digital Content, http://links.lww.com/QAI/C685). The reverse Weibull and Pareto underestimated this more than the Weibull model, particularly with PHIA-calibrated shape estimates (Figs. 2A–D).

In surveys with VLS >95%, all 3 models underestimated VL density <1000 copies/mL (Figs. 2E, F, see Figures S9 and S10, Supplemental Digital Content, http://links.lww.com/QAI/C685). Pareto and reverse Weibull underestimated low VL values more than the Weibull model, in these surveys. Increasing the shape parameter for Pareto and reverse Weibull reduced the underestimation for VL <1000 copies/mL in high-VLS surveys (see Figure S2, Supplemental Digital Content, http://links.lww.com/QAI/C685).

### Fréchet, Gamma, and Lognormal Models

**FIGURE 3.**
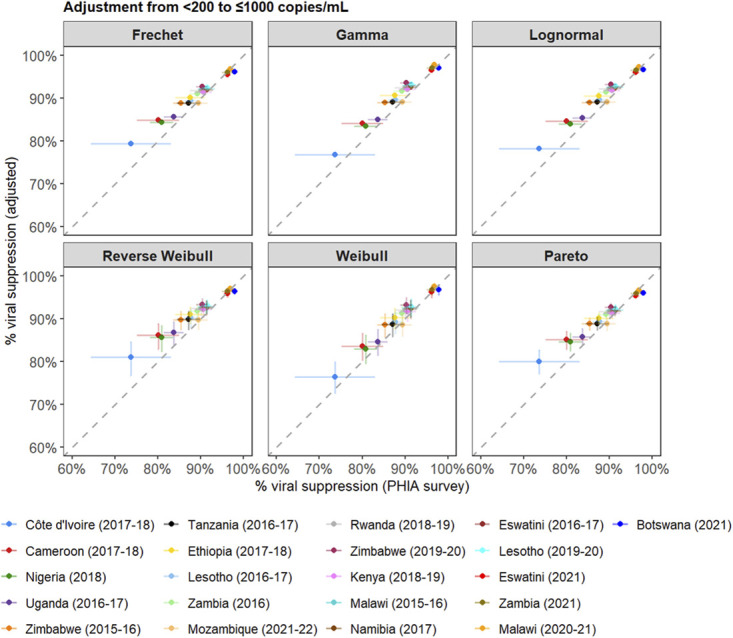
Scatter plots show the relationship between the observed percentage VLS estimates from the individual patient data in the PHIA surveys and the adjusted estimates (from <200 to ≤1000 copies/mL) using the Fréchet, gamma and lognormal model (parameters from calibration to the PHIA surveys), and the reverse Weibull, Weibull and Pareto models (using parameters from Johnson et al). Surveys in legend are sequenced in increasing order of observed VLS.

Table 2 reports the best fitting shape and SD parameters for these models. None improved predictions of VL ≤1000 copies/mL over the Weibull model using parameters from Johnson et al or PHIA recalibrated parameters, which performed best (Fig. 3, and see Figure S5, Supplemental Digital Content, http://links.lww.com/QAI/C685). RMSE for Fréchet, gamma, and lognormal models were lower than for reverse Weibull and Pareto models (using parameters from Johnson et al or recalibrated parameters) but similar to Weibull (Table 2).

**TABLE 2.** Shape Parameter Estimates and 95% Confidence Intervals for the Reverse Weibull, Weibull, Pareto, Fréchet, Gamma, and Lognormal Models Estimated From Calibration to the PHIA Survey Data

Model	Johnson et al	Pooled	Men	Women	15–24 yrs	25–34 yrs		35–44 yrs	45–54 yrs	55+ yrs
Reverse Weibull	2.81 (1.70–3.92)	2.98 (2.93–3.02)	2.86 (2.78–2.94)	3.04 (2.94–3.13)	2.46 (2.35–2.58)	2.52 (2.37–2.66)	2.84 (2.68–3.00)		3.81 (3.64–3.99)	4.32 (4.10–4.55)
Weibull	0.85 (0.43–1.26)	0.91 (0.90–0.92)	1.01 (0.98–1.03)	0.88 (0.85–0.90)	0.55 (0.53–0.56)	0.98 (0.97–0.99)	0.98 (0.97–0.99)		0.99 (0.98–1.02)	1.00 (0.98–1.04)
Pareto	1.73 (1.20–2.26)	—	—	—	—	—	—		—	—
Fréchet	—	1.86 (1.83–1.90)	1.94 (1.88–1.99)	1.84 (1.77–1.90)	1.52 (1.43–1.60)	1.69 (1.58–1.80)	1.88 (1.78–1.98)		2.13 (2.02–2.25)	2.27 (2.13–2.40)
Gamma	—	0.81 (0.79–0.84)	1.02 (0.97–1.07)	0.73 (0.69–0.79)	0.82 (0.74–0.90)	0.73 (0.65–0.83)	0.81 (0.73–0.90)		0.97 (0.87–1.08)	1.01 (0.89–1.14)
Lognormal	—	0.89 (0.88–0.90)	0.83 (0.80–0.85)	0.92 (0.90–0.95)	0.99 (0.94–1.03)	0.96 (0.90–1.02)	0.89 (0.83–0.94)		0.80 (0.75–0.86)	0.78 (0.72–0.83)

Plots of the VL density fits for the Fréchet, gamma, and lognormal models (see Figure S6, Supplemental Digital Content, http://links.lww.com/QAI/C685) closely match those of the Weibull model.

### Model Validation by Sex and Age

Using parameters from Johnson et al, prediction errors were slightly higher for men than women for Weibull, reverse Weibull, and Pareto models (see Figure S7, Supplemental Digital Content, http://links.lww.com/QAI/C685). The Weibull model had the lowest errors in both sexes, except for <50 to ≤1000 adjustment in men, where reverse Weibull performed best (see Table S3, Supplemental Digital Content, http://links.lww.com/QAI/C685). On average, in adjustment from <200 to ≤1000 using parameters from Johnson et al, the reverse Weibull model overestimated VLS by 2.6% (men) and 2.2% (women), Weibull by 1.2% and 1.5%, and Pareto by 1.8% and 1.5%, respectively.

By age, using parameters from Johnson et al, prediction errors were higher in younger age groups than older ones (see Figure S8, Supplemental Digital Content, http://links.lww.com/QAI/C685). The Weibull model consistently had the lowest errors among 15- to 24-year-olds and 25- to 34-year-olds, whereas all 3 models performed similarly for those aged 35+ years (see Table S3, Supplemental Digital Content, http://links.lww.com/QAI/C685). For adjustment from <200 to ≤1000, RMSE in 15- to 24-year-olds was 5.9% (reverse Weibull), 3.0% (Weibull) and 5.3% (Pareto); among those aged 55+ years, RMSE values were 2.0%, 1.8%, and 1.8%, respectively. On average, VLS was overestimated by 4.6% (reverse Weibull), 1.5% (Weibull), and by 3.7% (Pareto) in the youngest group, but by <1% in those aged 55+ years.

After recalibration to PHIAs, reverse Weibull shape estimates were similar by sex (men: 2.86; 95% confidence interval: 2.78–2.94; women 3.04; 2.94–3.13) but increased with age (Table 2). Weibull shape estimates were higher in men (1.01; 0.98–1.03) than women (0.88; 0.85–0.90), and lower among 15- to 24-year-olds but stable across older age groups (Table 2).

Using these sex- and age-specific parameters in the reverse Weibull model yielded similar prediction errors to parameters from Johnson et al for men, women, and individuals aged 35+ years (see Table S3, Supplemental Digital Content, http://links.lww.com/QAI/C685). Prediction errors for the reverse Weibull model were slightly lower for those younger than 35 years when using the age-specific shape estimated from the PHIAs. Adjustments using sex- and age-specific parameters for the Weibull model produced only marginal changes (Table 2, see Table S3, Supplemental Digital Content, http://links.lww.com/QAI/C685). For the Pareto model, using a shape of 1.20 reduced errors across most groups, except for those aged 45–54 and 55+ years, where errors were similar or higher compared with the shape estimate by Johnson et al.

On calibration to PHIAs, shape parameters for the Fréchet model were similar by sex (men: 1.94; 1.88–1.99; women: 1.84; 1.77–1.90) but varied by age, with higher shape with increasing age (Table 2). Gamma model estimates were higher in men (1.02; 0.97–1.07) than women (0.73; 0.69–0.79), similar among those younger than 45 years, and higher in those 45 years and older. For the lognormal model, SD was lower in men (0.83; 0.80–0.85) than in women (0.92; 0.90–0.95) and decreased with increasing age.

However, applying the Fréchet, gamma, or lognormal model to predict VL ≤1000 copies/mL by sex and age did not improve overall predictions compared with the Weibull (age/sex-indifferent) model (Table 2, see Tables S3–S6, Supplemental Digital Content, http://links.lww.com/QAI/C685).

In sensitivity analyses, restricting the analysis to PLHIV on ART for ≥12 months relied on ART duration data with substantial variability in missingness across the 14 PHIA surveys. Missingness for ART duration ranged from 6% in Eswatini (2016–2017) to 47% in Nigeria (2018) and was highest (>25%) in the 3 surveys with the lowest levels of VLS (see Table S8, Supplemental Digital Content, http://links.lww.com/QAI/C685). When refitting models to this ≥12-month subset (mean VLS: 87%), shape parameter estimates for the reverse Weibull, Weibull, Fréchet, and gamma distributions were significantly lower than those obtained from all 21 surveys (mean VLS: 89%), whereas lognormal shape estimates were largely unchanged (see Table S7, Supplemental Digital Content, http://links.lww.com/QAI/C685). However, when using the same 14 surveys but including all PLHIV on ART (mean VLS: 86%), significant differences in shape parameters were observed only for the reverse Weibull, Fréchet, and lognormal models, with Weibull and gamma estimates remaining stable (see Table S7, Supplemental Digital Content, http://links.lww.com/QAI/C685). Using shape estimates derived from the sensitivity analyses, VLS continued to be overestimated by all models in most surveys.

## DISCUSSION

We found that statistical models proposed by Johnson et al to adjust VLS reported at different thresholds to the global standard of ≤1000 copies/mL overestimated VLS for most national survey from sub-Saharan Africa, although point estimates remained within survey-based uncertainty ranges. Prediction errors were larger when adjusting from lower thresholds (<50) compared with higher thresholds (<200 or <400), and for surveys and subgroups with lower VLS (eg, men, younger individuals). Of 6 models explored, the Weibull model had lower prediction errors across all threshold and subgroups than the reverse Weibull and Pareto models and performed similarly to the Fréchet, gamma, and lognormal models. This contrasts the conclusion of Johnson et al^8^ to use the reverse Weibull model.

The overestimation of VLS by all 6 models and higher prediction errors in adjustments from lower thresholds is similar to validation results from Johnson et al.^8^ Likewise, Johnson et al found that shape parameters for the reverse Weibull and Pareto models were correlated with VLS. Specifically, they found higher shape parameter estimates for these models were estimated in regions with higher VLS levels and in adults with higher levels of VLS compared with children.^8^ Their Weibull estimates were similar across groups, aligning with our findings; however, although Johnson et al examined difference between children and adults, our analysis focused on variation among adult age groups.^8^ In contrast to our results where prediction errors were lowest for the Weibull model across all adjustments, in Johnson et al, the Weibull model only performed best on validation using the WHO drug resistance data at all thresholds, with similar performance across the 3 models on validation with the Brazilian national program data at all thresholds.^8^ This is related to the high VLS levels (>85%) reported across all timepoints in the Brazilian data,^8^ whereas in the WHO drug resistance data, the Weibull model had the lowest prediction errors for data from Honduras and Nicaragua with VLS of <75% using the ≤1000/mL threshold.^8^

Johnson et al^8^ noted that VL distributions among PLHIV on ART can vary substantially even when the fraction virally suppressed is the same. We found that the shape of the VL distribution among PLHIV on ART varied systematically with the fraction virally suppressed, with consistent discrepancies from our model distributions. In settings with high VLS (>95%), the distribution was right-skewed with a long tail, which was suitably modelled using the distributions we explored. In settings with lower VLS (<95%), the distribution had a slight hump of high VL values at the tail, which all models explored underestimated. Statistical distributions whose shape or SD parameters were correlated with VLS—reverse Weibull, Pareto, Fréchet, and lognormal—required higher shape (or lower SD) parameters to fit high VLS data but the reverse for low VLS settings. This explains why the reverse Weibull model, when calibrated to PHIAs with an average VLS of 89%, produced slightly higher, age-/sex-indifferent shape estimates compared with calibration to adult data by Johnson et al, where the average VLS was lower (85%). Furthermore, in low VLS settings, the reverse Weibull and Pareto models exhibited poor fit and larger prediction errors when higher shape parameters were used. A similar pattern was observed in our sensitivity analyses. Shape estimates for the reverse Weibull were lower when calibrating to the 14 pre-2020 PHIAs, which had slightly lower average VLS than the full set of 21 surveys, whereas the Weibull shape parameter remained comparatively stable. However, apparent differences in Weibull shape estimates—and in all distributions except the lognormal—when restricting to individuals on ART for ≥12 months are likely driven by data limitations in this subset. High levels of missing ART-duration data and smaller effective sample sizes in several surveys reduce precision and increase sensitivity to extreme observations, which can amplify differences in estimated shape parameters independent of any true underlying change in the VL distribution.

Our results suggest that the reverse Weibull model—recommended by the UNAIDS Reference Group on Estimates, Modelling, and Projections for standardizing VL measurements^8^—overestimates VLS more than the other 5 models in this analysis, particularly in low VLS settings. Adjustments using the reverse Weibull model in age/sex subgroups or countries with low VLS may lead to spuriously high levels of VLS, masking existing gaps in subgroups with suboptimal adherence or ART program challenges.^24^ Overestimation of VLS can lead to underestimation of HIV transmission and new infections in mathematical models like the UNAIDS-supported spectrum model where HIV transmission risk is informed by VLS levels.^25^

Although the Weibull model outperformed the reverse Weibull and Pareto and was comparable with the Fréchet, gamma, or lognormal models at adjusting VLS, it has limitations. First, it also fit poorly in low VLS settings, slightly overestimating VLS, especially for adjustments from the lowest (<50) threshold. Prediction errors for the Weibull model, like other models, were higher when adjusting from <50 to ≤1000 than from <200 or <400 to ≤1000. Conversely, because of its long upper tail, the Weibull model may predict too high VLs at the upper end of the distribution, limiting its use in modelling HIV transmission from PLHIV on ART.^8^ Although the empirical distribution of VLs among PLHIV on ART in low VLS settings appeared bimodal, we did not explore bimodal or mixture distributions as their parameters would likely correlate with VLS levels, risk overfitting sparse high-VL observations, and would not yield simple or practical adjustment functions for routine use. Although the gamma and lognormal models performed similarly to Weibull, they also lack simple closed-form mathematical adjustments, limiting practical use.

This study has several limitations. First, using data exclusively from sub-Saharan Africa may restrict the generalizability of our shape estimates, particularly for the reverse Weibull, Pareto, Fréchet, and lognormal models, to other regions. Second, our calibration relied on survey data, which may limit applicability of our results for adjusting estimates derived from routine programmatic data. Delays in processing routine VL specimens, as reported in some studies, can lead to inconsistent performance of VL assays at lower levels of viremia, potentially influencing VL distributions observed in routine program data.^26^

In conclusion, the Weibull model was more reliable for adjusting VLS estimates across thresholds than the recommended reverse Weibull model, which produced worse overestimation of VLS. Despite its limitations, the Weibull model's consistent shape parameter throughout most of the range of typical VL distributions, consistency in performance across subgroups (with only marginal differences when sex- or age-specific parameters are applied), and its mathematically simple adjustment make it the best currently available option for the UNAIDS-supported Spectrum model used for global epidemic estimation and evaluation of progress towards population-level VLS. However, all adjustment methods have the potential to introduce error, and efforts should be made to support countries in reporting using the recommended thresholds, to avoid reliance on adjustment methods.

## Supplementary Material
