# Supplementary material for: Validating HIV Viral Suppression Threshold Adjustments for Comparable Estimates Using Data From Nationally Representative Household Surveys in Sub-Saharan Africa
Source: J Acquir Immune Defic Syndr. 2026 Apr 15;101(8):827–35. doi: 10.1097/QAI.0000000000003878 (PMC13372362; doi:10.1097/QAI.0000000000003878)

# Supplementary information

**Table S1A**. Total number, age range, lower limit of HIV RNA detection and survey weighted VLS estimates and 95% confidence interval for people living with HIV on antiretroviral therapy included from each PHIA survey. Corresponding counts of PLHIV on ART with viral load <50, <200, <400, and <1000 copies/mL are provided in Table S1B.

| Survey | PLHIV on ART | Survey age range | Lower limit of HIV RNA detection reported for PLHIV in survey (number of PLHIV reported at threshold) | Survey VLS (% ≤1000 copies/mL) (95% CI) |
| --- | --- | --- | --- | --- |
| Botswana (2021) | 3208 | 15-64 | TND (1775), <LLOQ: 20 (519), <LLOQ: 40 (34), <LLOQ: 400 (13) | 97.9 (97.2, 98.6) |
| Cameroon (2017-2018) | 493 | 15-64 | TND (313), <LLOQ: 40 (11), <LLOQ: 839 (2) | 80.1 (75.1, 85.0) |
| Côte d'Ivoire (2017-2018) | 207 | 15-64 | TND (52), <LLOQ: 20 (32) | 73.7 (64.3, 83.0) |
| Eswatini (2016-2017) | 2369 | 15+ | TND (1220), <LLOQ: 20 (460), <LLOQ: 40 (33), <LLOQ: 839 (5) | 91.4 (90.3, 92.5) |
| Eswatini (2021) | 2660 | 15+ | TND (1457), <LLOQ: 20 (541), <LLOQ: 40 (30), <LLOQ: 400 (10) | 96.2 (95.4, 97.0) |
| Ethiopia (2017-2018) | 476 | 15-64 | TND (240), <LLOQ: 20 (61), <LLOQ: 40 (1), <LLOQ: 839 (1) | 87.6 (84.1, 91.0) |
| Kenya (2018-2019) | 1176 | 15-64 | TND (497), <LLOQ: 20 (273), <LLOQ: 40 (3), <LLOQ: 400 (4) | 90.6 (88.6, 92.6) |
| Lesotho (2016-2017) | 2435 | 15-59 | TND (1239), <LLOQ: 20 (402), <LLOQ: 400 (15) | 87.7 (86.1, 89.3) |
| Lesotho (2019-2020) | 3260 | 15+ | TND (1875), <LLOQ: 20 (406), <LLOQ: 40 (13), <LLOQ: 400 (10) | 91.5 (90.3, 92.6) |
| Malawi (2015-2016) | 1564 | 15-64 | TND (1156), <LLOQ: 40 (176) | 91.3 (89.3, 93.3) |
| Malawi (2020-2021) | 2167 | 15+ | TND (1712), <LLOQ: 40 (255), <LLOQ: 80 (43), <LLOQ: 839 (8) | 96.9 (96.0, 97.7) |
| Mozambique (2021-2022) | 1482 | 15+ | TND (622), <LLOQ: 20 (198), <LLOQ: 40 (14), <LLOQ: 400 (4) | 89.4 (87.3, 91.6) |
| Namibia (2017) | 2069 | 15-64 | TND (966), <LLOQ: 20 (343), <LLOQ: 40 (4), <LLOQ: 400 (5) | 91.3 (89.8, 92.9) |
| Nigeria (2018) | 1322 | 15-64 | TND (435), <LLOQ: 20 (250), <LLOQ: 40 (13), <LLOQ: 400 (5) | 80.9 (78.2, 83.6) |
| Rwanda (2018-2019) | 767 | 15-64 | TND (368), <LLOQ: 20 (165), <LLOQ: 40 (2) | 90.1 (87.6, 92.6) |
| Tanzania (2016-2017) | 1022 | 15+ | TND (477), <LLOQ: 20 (182), <LLOQ: 40 (3), <LLOQ: 400 (4) | 87.2 (84.5, 89.8) |
| Uganda (2016-2017) | 1185 | 15-64 | TND (470), <LLOQ: 20 (160) | 83.7 (81.4, 85.9) |
| Zambia (2016) | 1556 | 15-59 | TND (827), <LLOQ: 20 (281), <LLOQ: 550 (1) | 89.2 (87.4, 91.1) |
| Zambia (2021) | 1906 | 15+ | TND (1244), <LLOQ: 20 (254), <LLOQ: 400 (4) | 96.3 (95.0, 97.5) |
| Zimbabwe (2015-2016) | 2497 | 15+ | TND (1354), <LLOQ: 20 (418), <LLOQ: 182 (2) | 85.4 (83.6, 87.2) |
| Zimbabwe (2019-2020) | 2547 | 15+ | TND (1599), <LLOQ: 20 (367), <LLOQ: 40 (15), <LLOQ: 400 (3) | 90.3 (89.0, 91.7) |

TND – Target not detected, <LLOQ: 20 - less than lower limit of quantification of 20, <LLOQ: 40 - less than lower limit of quantification of 40, <LLOQ: 80 - less than lower limit of quantification of 80, <LLOQ: 182 - less than lower limit of quantification of 182, <LLOQ: 400 - less than lower limit of quantification of 400, <LLOQ: 550 - less than lower limit of quantification of 550, <LLOQ: 839 - less than lower limit of quantification of 839.

**Table S1B:** PLHIV on ART with viral loads <50, <200, <400, and <1000 copies/mL, and survey‑weighted viral load suppression estimates with 95% confidence intervals by survey.

|  | **Count of PLHIV on ART** | | | | | **Survey weighted VLS estimates** | | | |
| --- | --- | --- | --- | --- | --- | --- | --- | --- | --- |
| **Survey** | **PLHIV on ART** | **VL<50** | **VL <200** | **VL <400** | **VL ≤1000** | **% 50 copies/mL**  **(95% CI)** | **% <200 copies/mL (95% CI)** | **% <400 copies/mL (95% CI)** | **% ≤1000 copies/mL (95% CI)** |
| Botswana (2021) | 3208 | 2672 | 3027 | 3092 | 3142 | 81.2 (78.6, 83.8) | 93.7 (92.2, 95.2) | 96.1 (94.9, 97.2) | 97.9 (97.2, 98.6) |
| Cameroon (2017-18) | 493 | 329 | 369 | 380 | 391 | 68.4 (62.7, 74.1) | 76.4 (71.3, 81.6) | 78.3 (73.2, 83.4) | 80.1 (75.1, 85.0) |
| Côte d'Ivoire (2017-2018) | 207 | 111 | 149 | 153 | 159 | 53.7 (42.1, 65.2) | 68.4 (57.4, 79.4) | 70.5 (60.7, 80.3) | 73.7 (64.3, 83.0) |
| Eswatini (2016-17) | 2369 | 1898 | 2084 | 2135 | 2169 | 79.3 (77.4, 81.1) | 87.5 (86.1, 88.8) | 89.9 (88.7, 91.0) | 91.4 (90.3, 92.5) |
| Eswatini (2021) | 2660 | 2279 | 2479 | 2519 | 2563 | 85.2 (83.5, 86.9) | 92.8 (91.5, 94.0) | 94.5 (93.5, 95.4) | 96.2 (95.4, 97.0) |
| Ethiopia (2017-18) | 476 | 345 | 398 | 410 | 414 | 72.1 (67.3, 76.8) | 84.4 (80.7, 88.1) | 86.7 (83.4, 90.0) | 87.6 (84.1, 91.0) |
| Kenya (2018-19) | 1176 | 889 | 1007 | 1033 | 1065 | 75.0 (71.8, 78.1) | 86.3 (83.8, 88.8) | 88.0 (85.6, 90.4) | 90.6 (88.6, 92.6) |
| Lesotho (2016-17) | 2435 | 1849 | 2031 | 2071 | 2136 | 75.6 (73.8, 77.5) | 83.2 (81.5, 84.9) | 84.9 (83.2, 86.6) | 87.7 (86.1, 89.3) |
| Lesotho (2019-20) | 3260 | 2599 | 2885 | 2932 | 2997 | 78.6 (76.9, 80.3) | 87.8 (86.5, 89.1) | 89.3 (88.1, 90.6) | 91.5 (90.3, 92.6) |
| Malawi (2015-16) | 1564 | 1336 | 1377 | 1390 | 1428 | 84.4 (81.9, 87.0) | 87.5 (85.2, 89.7) | 88.5 (86.3, 90.8) | 91.3 (89.3, 93.3) |
| Malawi (2020-21) | 2167 | 1978 | 2055 | 2077 | 2099 | 91.1 (89.6, 92.6) | 94.8 (93.7, 95.9) | 95.8 (94.8, 96.8) | 96.9 (96.0, 97.7) |
| Mozambique (2021-22) | 1482 | 1022 | 1257 | 1307 | 1349 | 66.0 (63.0, 68.9) | 82.4 (79.7, 85.1) | 86.1 (83.9, 88.4) | 89.4 (87.3, 91.6) |
| Namibia (2017) | 2069 | 1550 | 1782 | 1829 | 1867 | 75.9 (73.4, 78.4) | 87.0 (85.1, 89.0) | 89.3 (87.6, 91.1) | 91.3 (89.8, 92.9) |
| Nigeria (2018) | 1322 | 842 | 991 | 1028 | 1066 | 62.7 (59.3, 66.1) | 75.7 (72.8, 78.6) | 78.3 (75.4, 81.1) | 80.9 (78.2, 83.6) |
| Rwanda (2018-19) | 767 | 616 | 673 | 683 | 697 | 79.5 (76.5, 82.4) | 86.8 (84.0, 89.6) | 88.1 (85.4, 90.8) | 90.1 (87.6, 92.6) |
| Tanzania (2016-17) | 1022 | 750 | 838 | 861 | 890 | 73.3 (69.5, 77.1) | 82.4 (79.3, 85.6) | 84.4 (81.4, 87.5) | 87.2 (84.5, 89.8) |
| Uganda (2016-17) | 1185 | 742 | 904 | 952 | 983 | 63.5 (60.4, 66.5) | 77.5 (74.9, 80.2) | 81.4 (79.1, 83.7) | 83.7 (81.4, 85.9) |
| Zambia (2016) | 1556 | 1236 | 1337 | 1366 | 1394 | 79.5 (77.0, 82.0) | 85.7 (83.5, 88.0) | 87.6 (85.5, 89.7) | 89.2 (87.4, 91.1) |
| Zambia (2021) | 1906 | 1640 | 1773 | 1800 | 1825 | 87.8 (85.7, 89.8) | 93.6 (92.1, 95.1) | 94.8 (93.4, 96.2) | 96.3 (95.0, 97.5) |
| Zimbabwe (2015-16) | 2497 | 1943 | 2089 | 2132 | 2166 | 76.4 (74.4, 78.5) | 82.4 (80.5, 84.2) | 84.1 (82.3, 86.0) | 85.4 (83.6, 87.2) |
| Zimbabwe (2019-20) | 2547 | 2142 | 2264 | 2283 | 2313 | 83.6 (81.9, 85.4) | 88.4 (86.8, 89.9) | 89.0 (87.5, 90.6) | 90.3 (89.0, 91.7) |

**Table S2.** Number of countries reporting viral load suppression data to UNAIDS at different thresholds for 2015 to 2023.

|  | Threshold for reporting viral load suppression in copies/mL | | | |
| --- | --- | --- | --- | --- |
| Year (number of reporting countries) | <50 | <200 | <400 | <1000 |
| 2015 (27) | 0 | 5 | 0 | 22 |
| 2016 (46) | 0 | 7 | 0 | 39 |
| 2017 (69) | 0 | 11 | 0 | 58 |
| 2018 (91) | 0 | 11 | 0 | 80 |
| 2019 (90) | 1 | 10 | 0 | 79 |
| 2020 (98) | 2 | 11 | 1 | 84 |
| 2021 (98) | 2 | 13 | 1 | 82 |
| 2022 (96) | 2 | 11 | 1 | 82 |
| 2023 (91) | 2 | 10 | 1 | 78 |

Source: UNAIDS Estimates 2024 Spectrum files (<https://hivtools.unaids.org/spectrum-file-request/>)


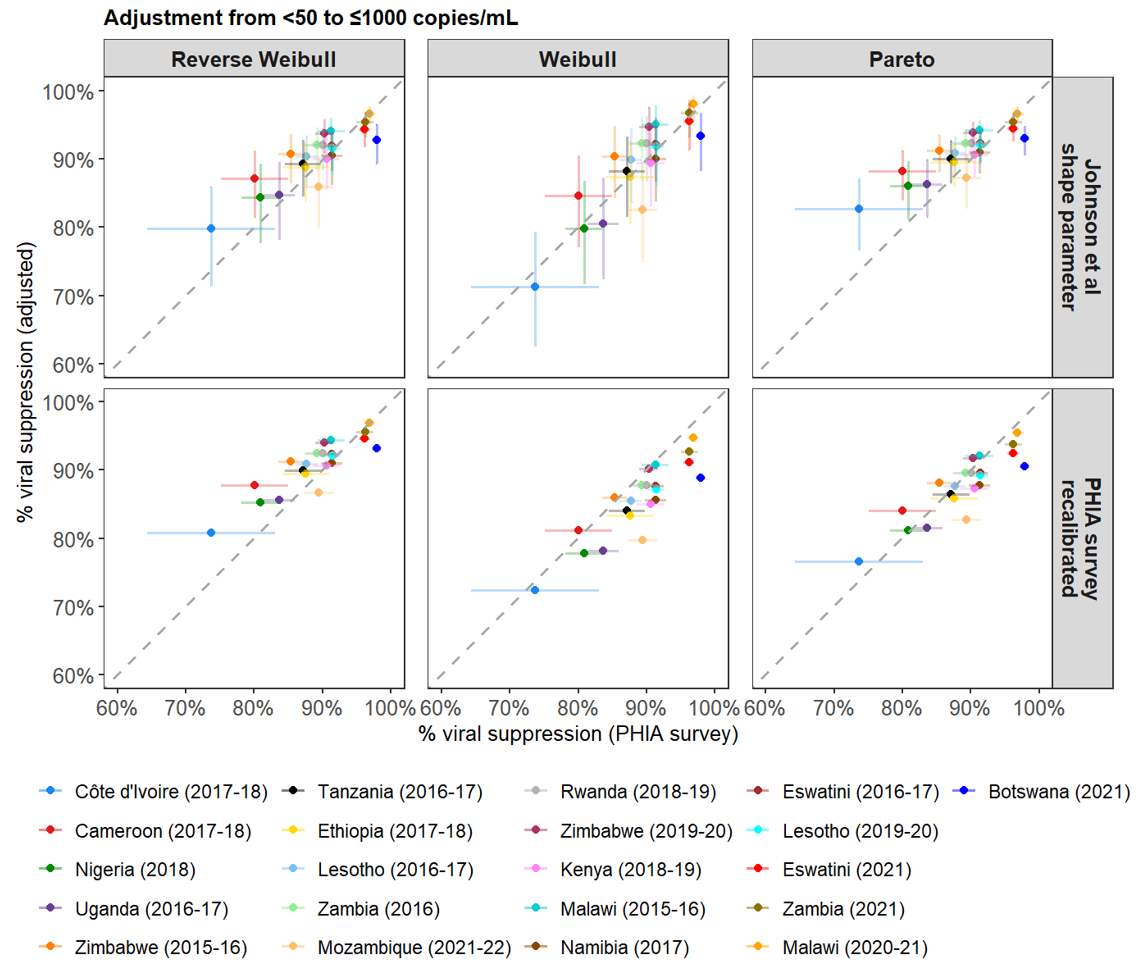


**Figure S1A.** Scatter plots show the relationship between the observed percentage VLS estimates from the individual patient data in the PHIA surveys and the adjusted estimates (from <50 to ≤1000 copies/mL) using the reverse Weibull, Weibull and Pareto models and parameters from Johnson et al and calibration to PHIA survey data. Shape parameter for the Pareto model on calibration to PHIA survey data used in Figure is 1.20. Surveys in legend are sequenced in increasing order of observed VLS.


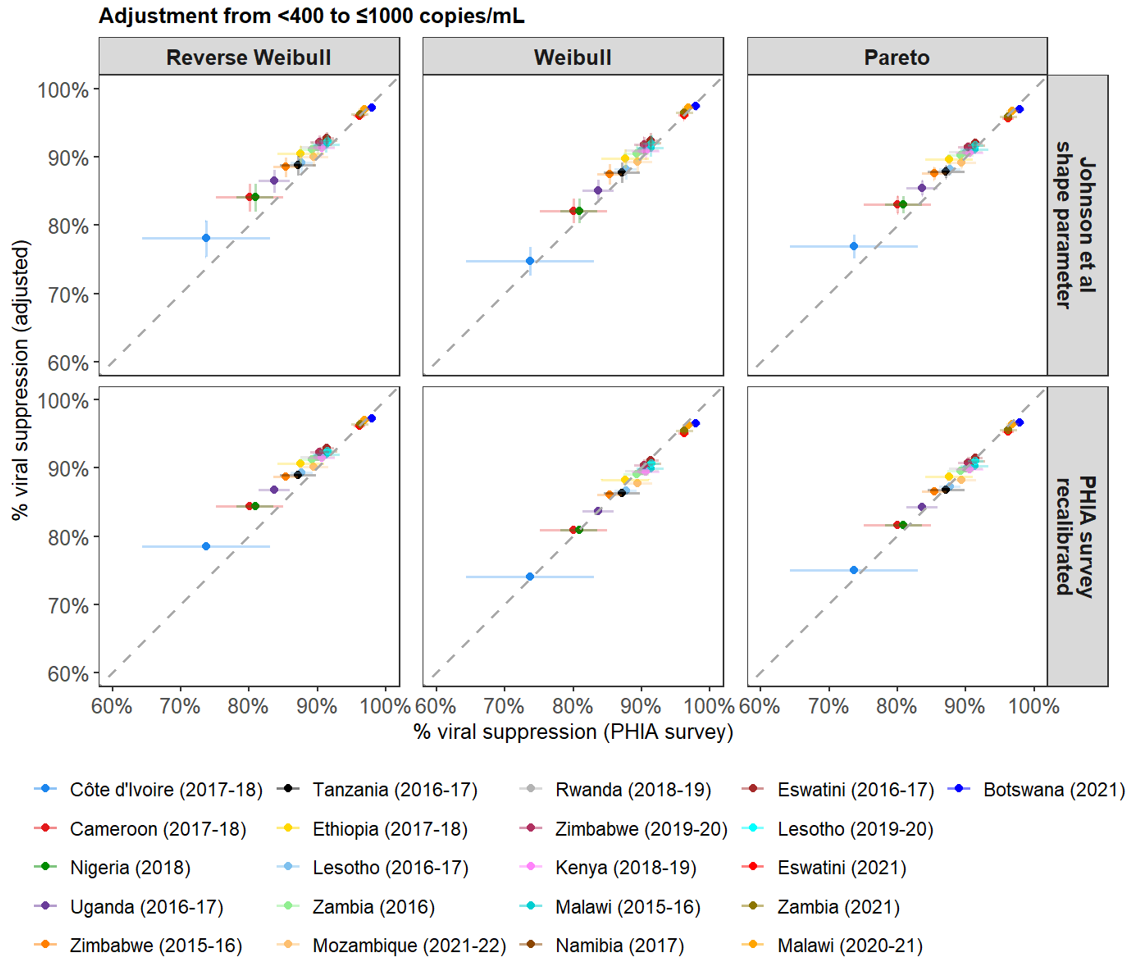


**Figure S1B.** Scatter plots show the relationship between the observed percentage VLS estimates from the individual patient data in the PHIA surveys and the adjusted estimates (from <400 to ≤1000 copies/mL) using the reverse Weibull, Weibull and Pareto models and parameters from Johnson et al and calibration to PHIA survey data. Shape parameter for the Pareto model on calibration to PHIA survey data used in Figure is 1.20. Surveys in legend are sequenced in increasing order of observed VLS.

**Appendix S1. Modelling viral loads among PLHIV on ART using the Pareto distribution**

The Pareto distribution was not suitable for likelihood-based fitting to individual-level survey observations because scale parameter $m$ defines the lower limit of the distribution. However, we explored a range of plausible shape parameter values and assessed their visual fits to the PHIA data and how they performed in predicting the proportion with VL ≤1000 copies from the observed proportions <50, <200 or <400 copies/mL.

Plots of the empirical distribution of viral loads observed from the PHIA surveys and probability density estimates for the Pareto distribution using the shape parameter from Johnson *et al.* (shape = 1.73) showed that the Pareto model overestimates the probability of VLs being <1000 copies/mL in surveys with VLS <90% and underestimates this probability in surveys with VLS >90% **(Figure S2)**. A lower shape parameter value (shape = 1.20) improved the fit of the Pareto model to the empirical data in surveys with VLS <90% (**Figure S2A, S2B and S2C**) but increased the underestimation of the probability of VLs being <1000 copies/mL in surveys with VLS >90% **(Figure S2)**. Using higher shape parameter values (e.g. shape ≥ 2.00) compared to estimates from Johnson *et al.* improved the fits of the Pareto distribution to the observed data in surveys with VLS >90% but increased the overestimation of the probability of VLs being ≤1000 copies/mL in surveys with VLS <90% **(Figure S2)**.

The modelled lower limit of the Pareto distribution, which is scale parameter dependent, was in some cases higher than the lower bound of VL values among PLHIV on ART in the PHIA survey data. For example, when using the Pareto distribution and shape parameter from Johnson *et al.* to model 74% of VL values being ≤1000 copies/mL (observed in the Cote d’Ivoire, 2017-2018 survey), the scale parameter required translates to a lower bound VL value of 24 copies/mL, which was greater than the lower limit of detection (20 copies/mL) in the survey. To model lower levels of viral load suppression using the Pareto distribution required even larger scale parameters which translate to even greater lower bound values which exceed the lower limit observed in the empirical data **(Figure S4)**.

Figure S4 shows that for different shape parameter values for the Pareto distribution, the modelled VLS (VL ≤1000 copies/mL) at which the lower bound of the Pareto distribution would exceed the lower limit of VL (20 copies/mL) in the empirical data varies. For shape parameter values of 1.20, 1.73, 2.00 and 2.50, modelling ≤63%, ≤76%, ≤81% and ≤88% of VL values being ≤1000 copies/mL results in a lower bound VL value of ≥20 copies/mL **(Figure S4)**.

**
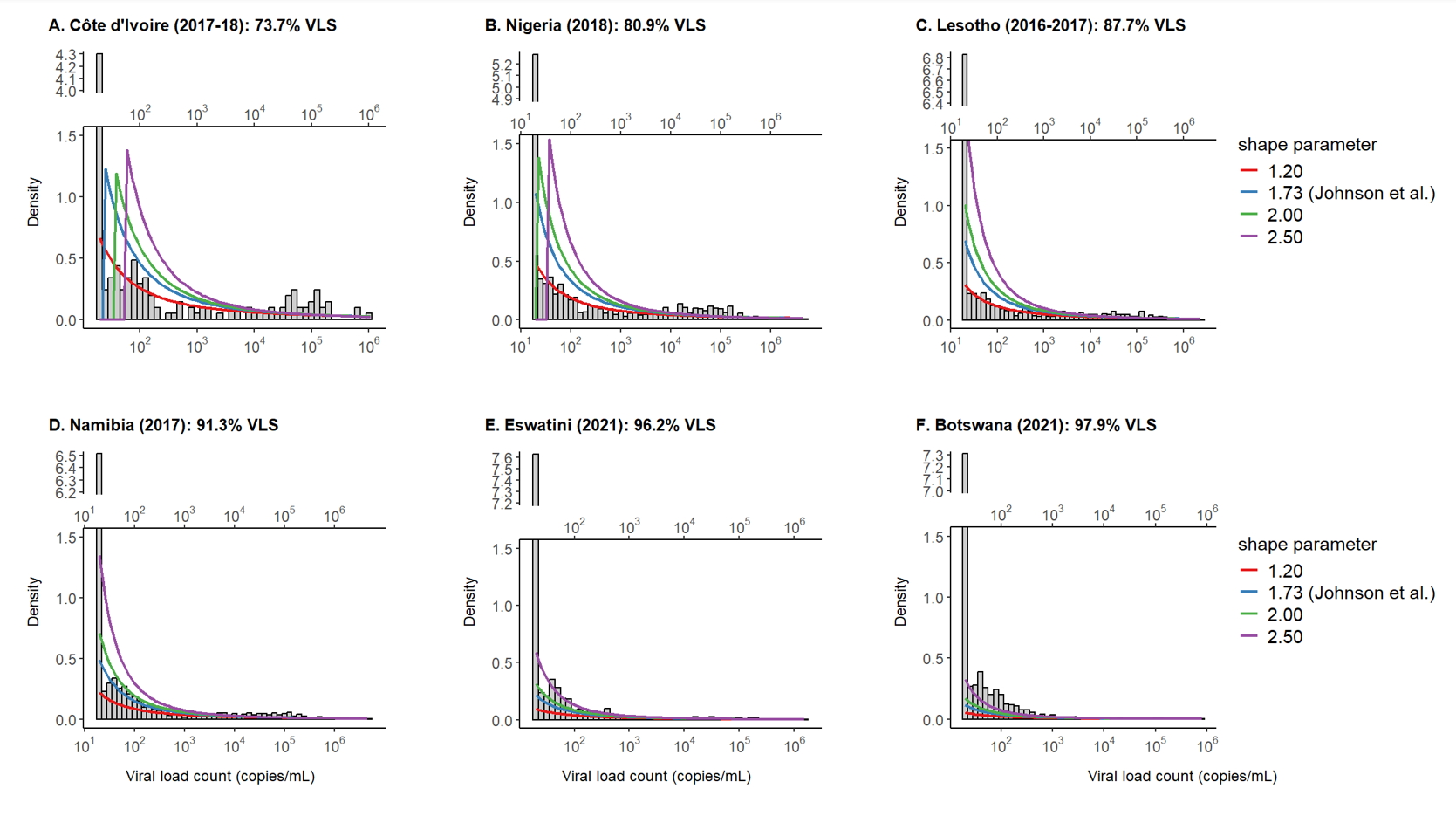
**

**Figure S2.** Histograms show the distribution of observed viral loads among PLHIV on ART in the A. Côte d'Ivoire (2017-18); B. Nigeria (2018); C. Lesotho (2016-2017); D. Namibia (2018); E. Eswatini (2021) and F. Botswana (2021) PHIA surveys. Lines show the probability density estimates for the Pareto distribution using shape parameters from Johnson *et al* (dashed line) and other plausible values*.* Note: the scale parameters were set so the cumulative probability of a viral load ≤1000 copies/mL is the same as VLS estimated from the survey data. Breaks in the y‑axis were included to allow clearer visualisation of the distribution.

**
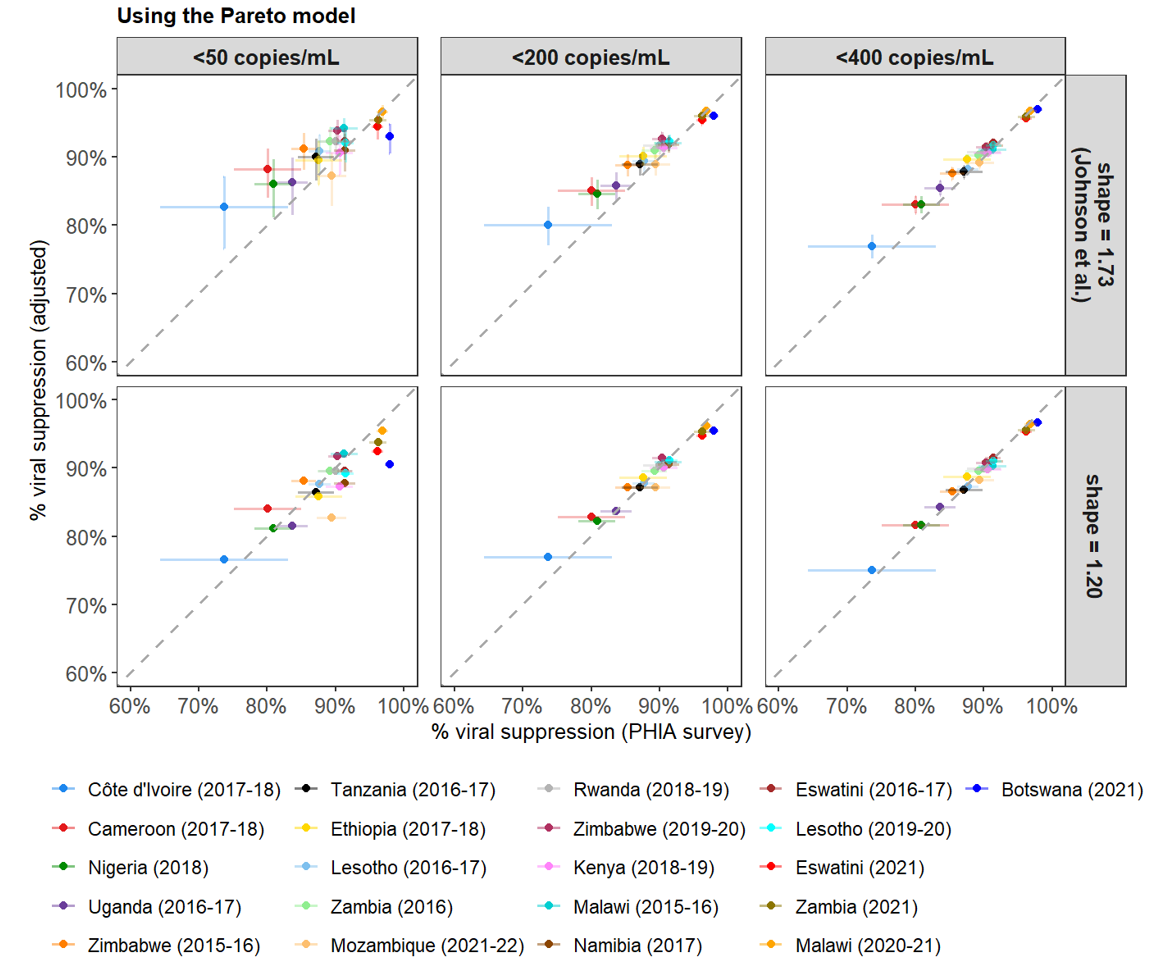
**

**Figure S3A** Scatter plots show the relationship between the observed percentage VLS estimates from the individual patient data in the PHIA surveys and the adjusted estimates (from <50, <200 and <400 to ≤1000 copies/mL) using the Pareto model and parameters from Johnson et al (shape = 1.73) or shape = 1.20. Root-mean-squared error (RMSE) when using the Pareto model with shape parameter 1.73 vs. 1.20 (RMSE for adjustment from <50 to ≤1000: 3.8% vs. 3.0%; RMSE for <200 to ≤1000: 2.5% vs. 1.4% and RMSE for <400 to ≤1000: 1.4% vs. 0.8%). Surveys in legend are sequenced in increasing order of observed VLS.


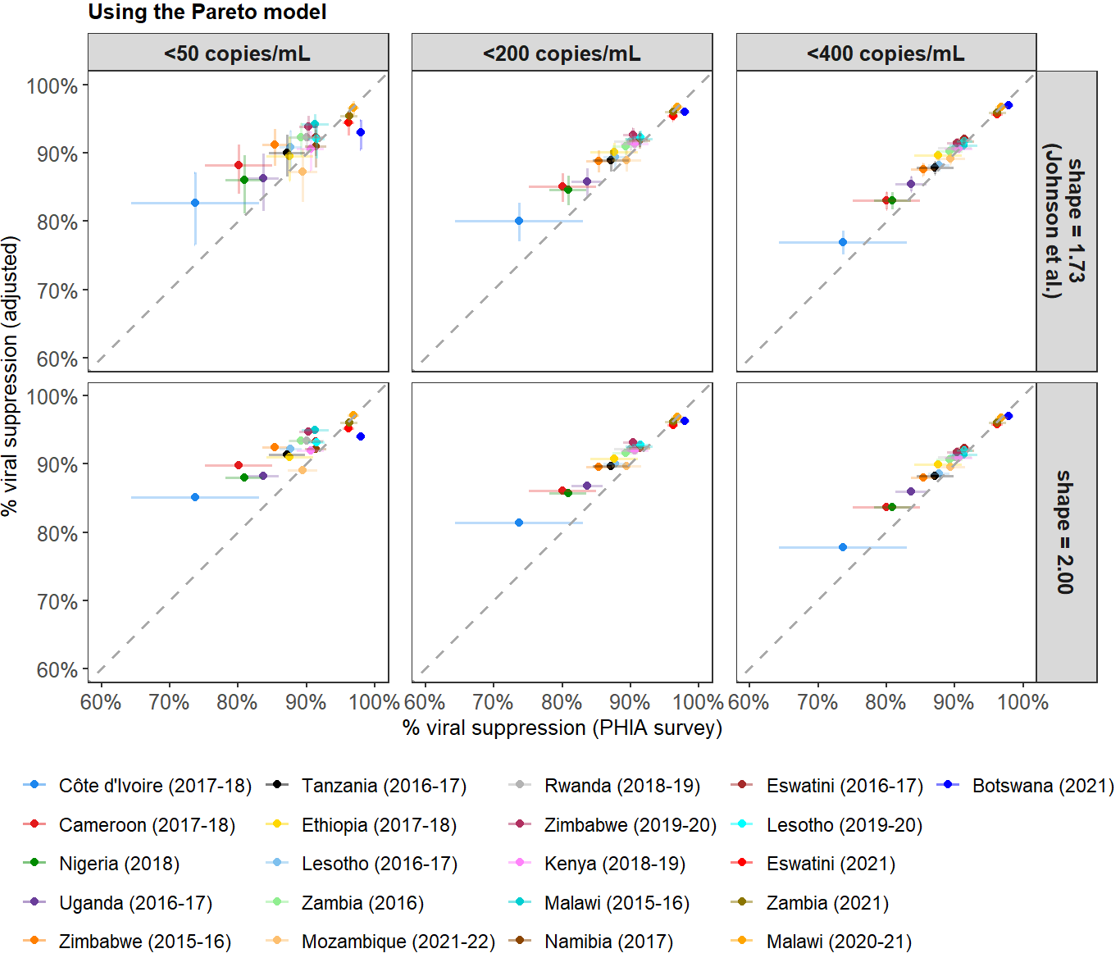


**Figure S3B.** Scatter plots show the relationship between the observed percentage VLS estimates from the individual patient data in the PHIA surveys and the adjusted estimates (from <50, <200 and <400 to ≤1000 copies/mL) using the Pareto model and parameters from Johnson et al (shape = 1.73) or shape = 2.00. Root-mean-squared error (RMSE) when using the Pareto model with shape parameter 1.73 vs. 2.00 (RMSE for adjustment from <50 to ≤1000: 3.8% vs. 4.8%; RMSE for <200 to ≤1000: 2.5% vs. 3.1% and RMSE for <400 to ≤1000: 1.4% vs. 1.8%). Surveys in legend are sequenced in increasing order of observed VLS.


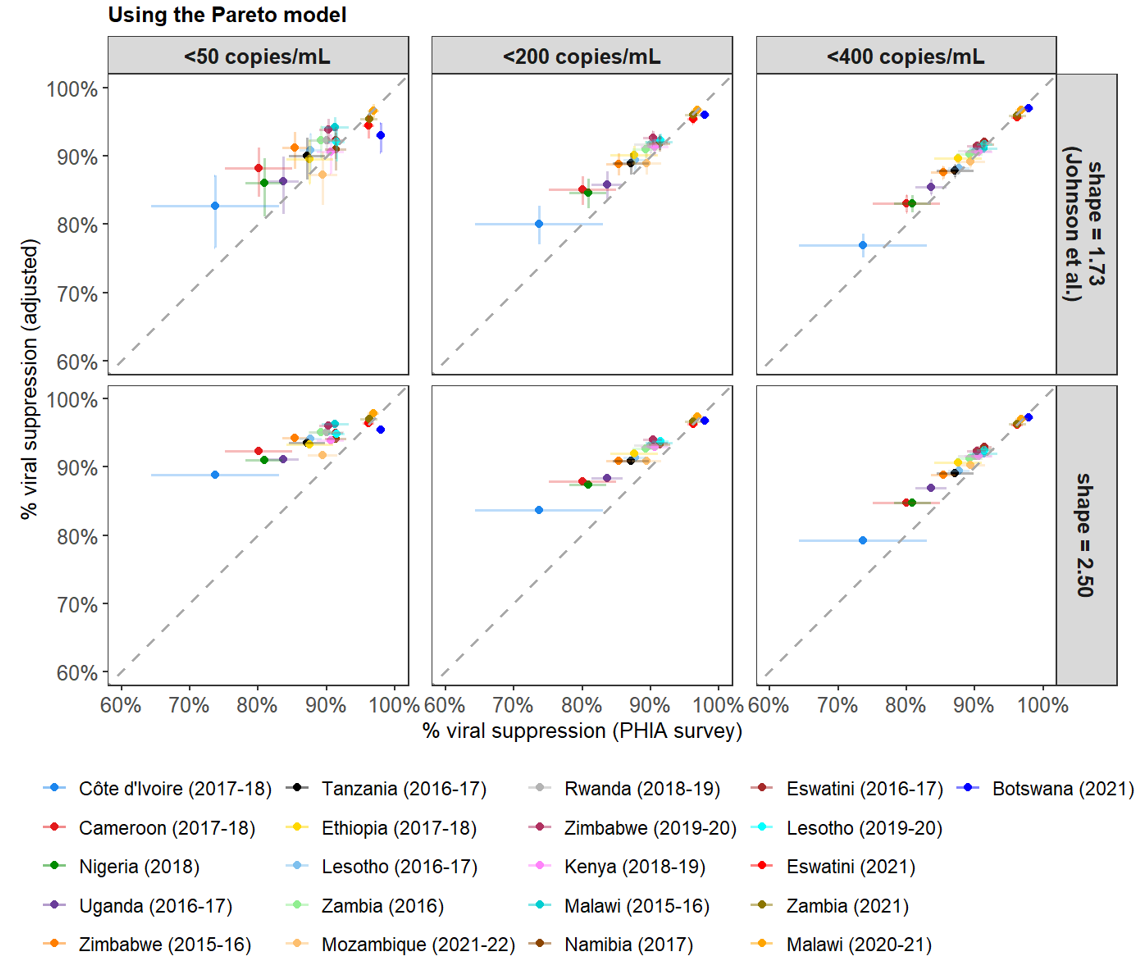


**Figure S3C.** Scatter plots show the relationship between the observed percentage VLS estimates from the individual patient data in the PHIA surveys and the adjusted estimates (from <50, <200 and <400 to ≤1000 copies/mL) using the Pareto model and parameters from Johnson et al (shape = 1.73) or shape = 2.50. Root-mean-squared error (RMSE) when using the Pareto model with shape parameter 1.73 vs. 2.50 (RMSE for adjustment from <50 to ≤1000: 3.8% vs. 6.6%; RMSE for <200 to ≤1000: 2.5% vs. 4.2% and RMSE for <400 to ≤1000: 1.4% vs. 2.5%). Surveys in legend are sequenced in increasing order of observed VLS.

**
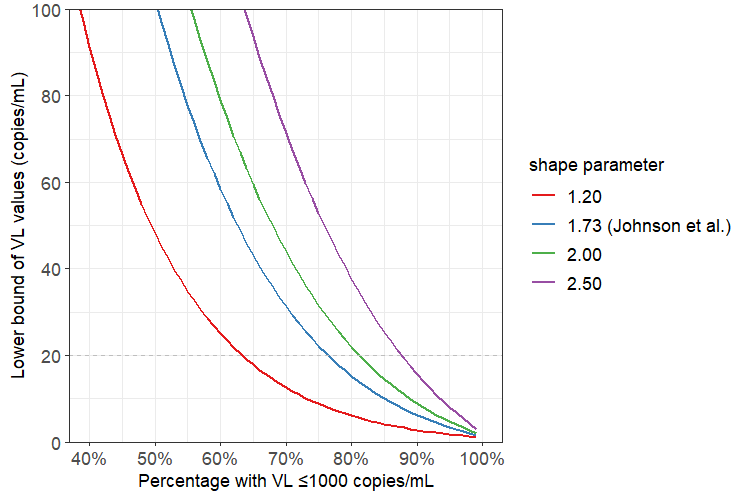
**

**Figure S4.** Plot shows the relationship between the lower bound (${10}^{m}$), where $m$ is the scale parameter, and the proportion ≤1000 copies/mL for the Pareto distribution using different shape parameter values.


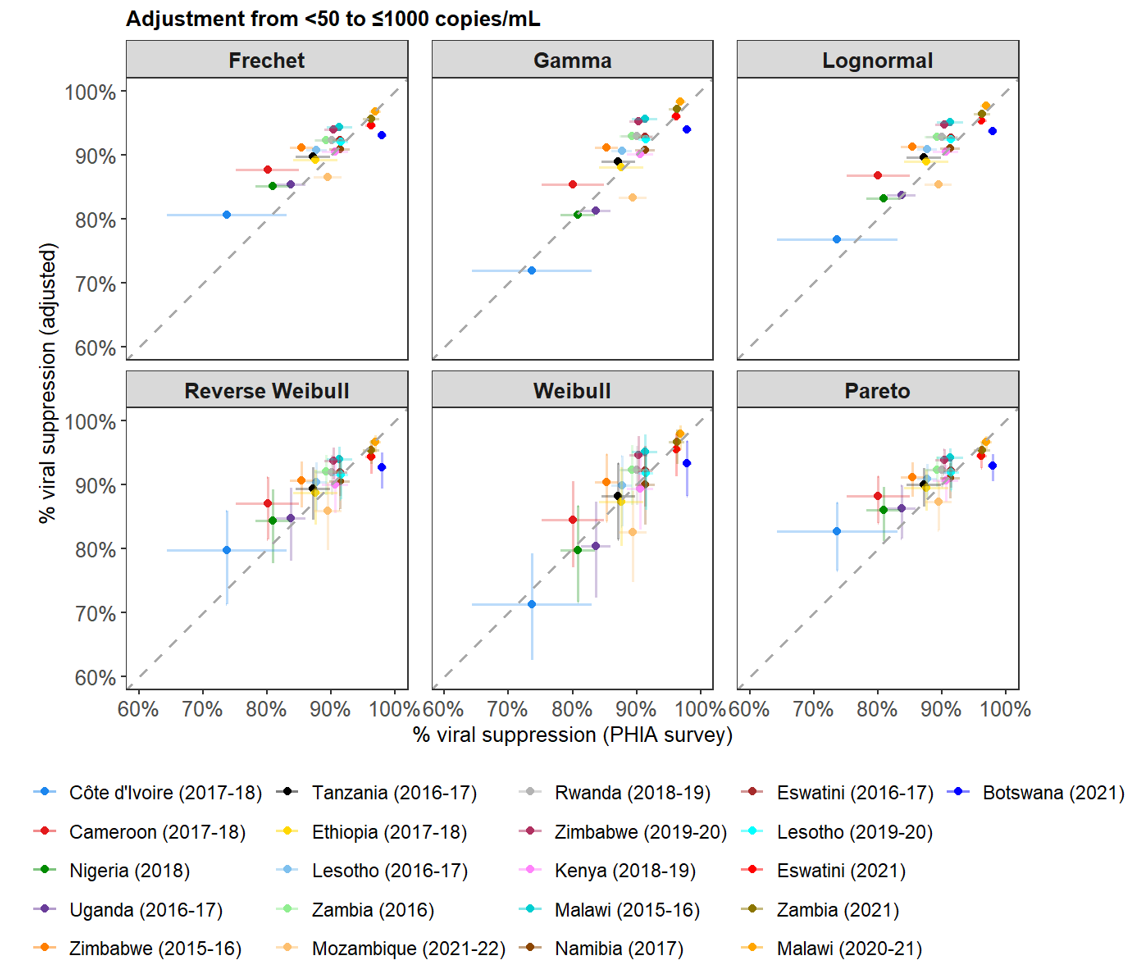


**Figure S5A.** Scatter plots show the relationship between the observed percentage VLS estimates from the individual patient data in the PHIA surveys and the adjusted estimates (from <50 to ≤1000 copies/mL) using the Fréchet, gamma and lognormal model (parameters from calibration to the PHIA surveys), and the reverse Weibull, Weibull and Pareto models (using parameters from Johnson *et al*.). Surveys in legend are sequenced in increasing order of observed VLS.

**
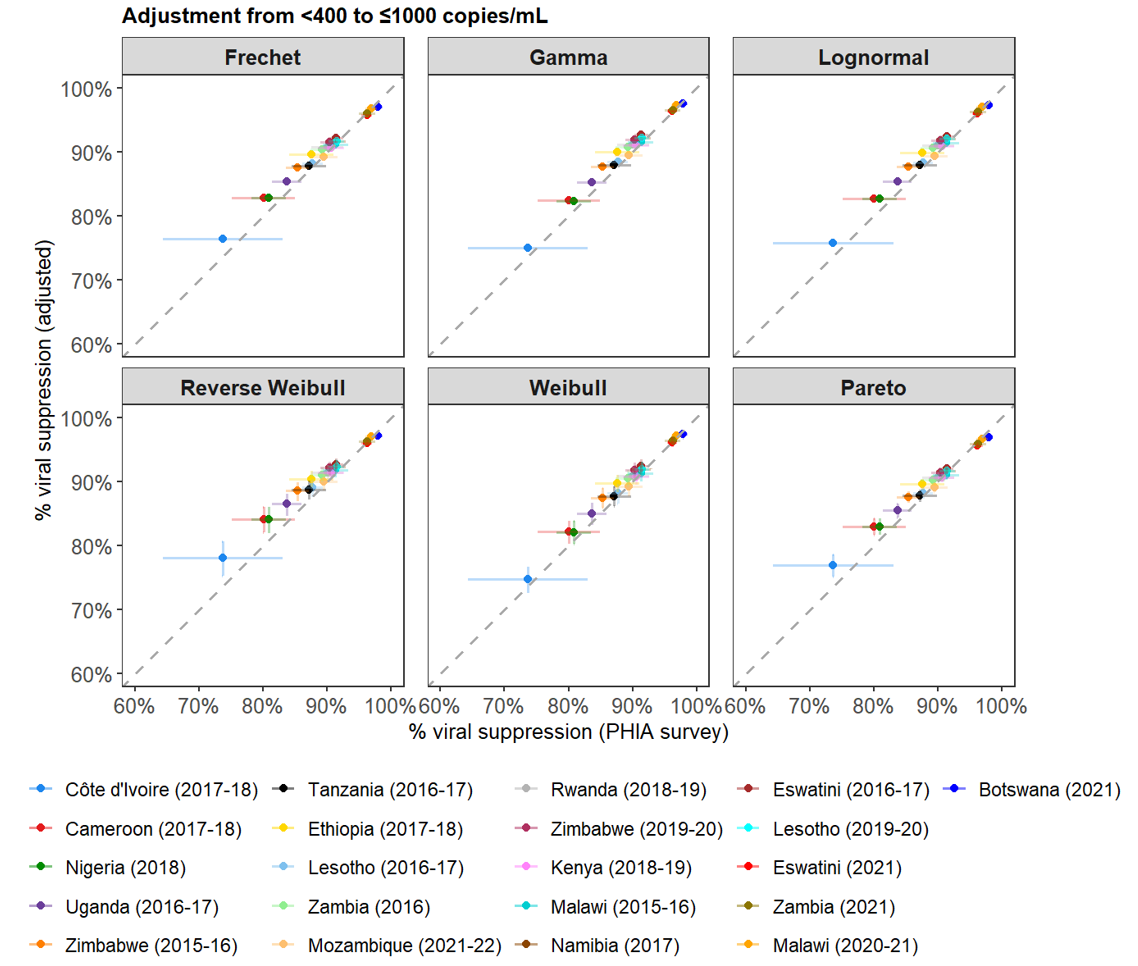
**

**Figure S5B.** Scatter plots show the relationship between the observed percentage VLS estimates from the individual patient data in the PHIA surveys and the adjusted estimates (from <400 to ≤1000 copies/mL) using the Fréchet, gamma and lognormal model (parameters from calibration to the PHIA surveys), and the reverse Weibull, Weibull and Pareto models (using parameters from Johnson *et al*.). Surveys in legend are sequenced in increasing order of observed VLS.


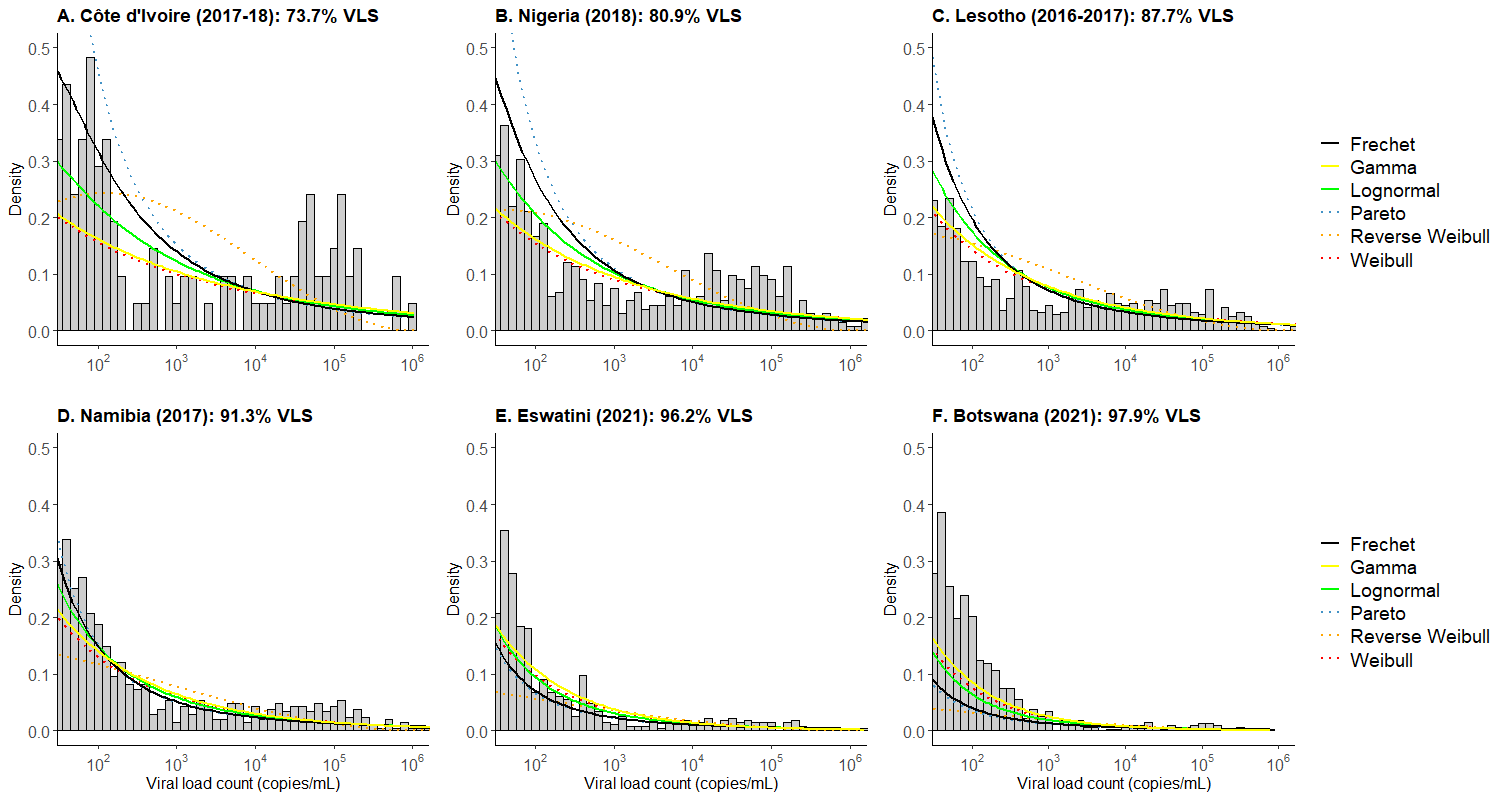


**Figure S6.** Histograms show the distribution of observed viral loads among PLHIV on ART in the A. Côte d'Ivoire (2017-18); B. Nigeria (2018); C. Lesotho (2016-2017); D. Namibia (2018); E. Eswatini (2021) and F. Botswana (2021) PHIA surveys. Lines show the probability density estimates for the Fréchet, gamma, lognormal using shape parameters from calibration to PHIA surveys (solid lines), and Pareto, reverse Weibull and Weibull models using shape parameters from Johnson *et al* (dotted lines). Note: the scale parameters were set so the cumulative probability of a viral load ≤1000 copies/mL is the same as VLS estimated from the survey data. The x-axis was truncated at 50 copies/mL for visualization purposes.


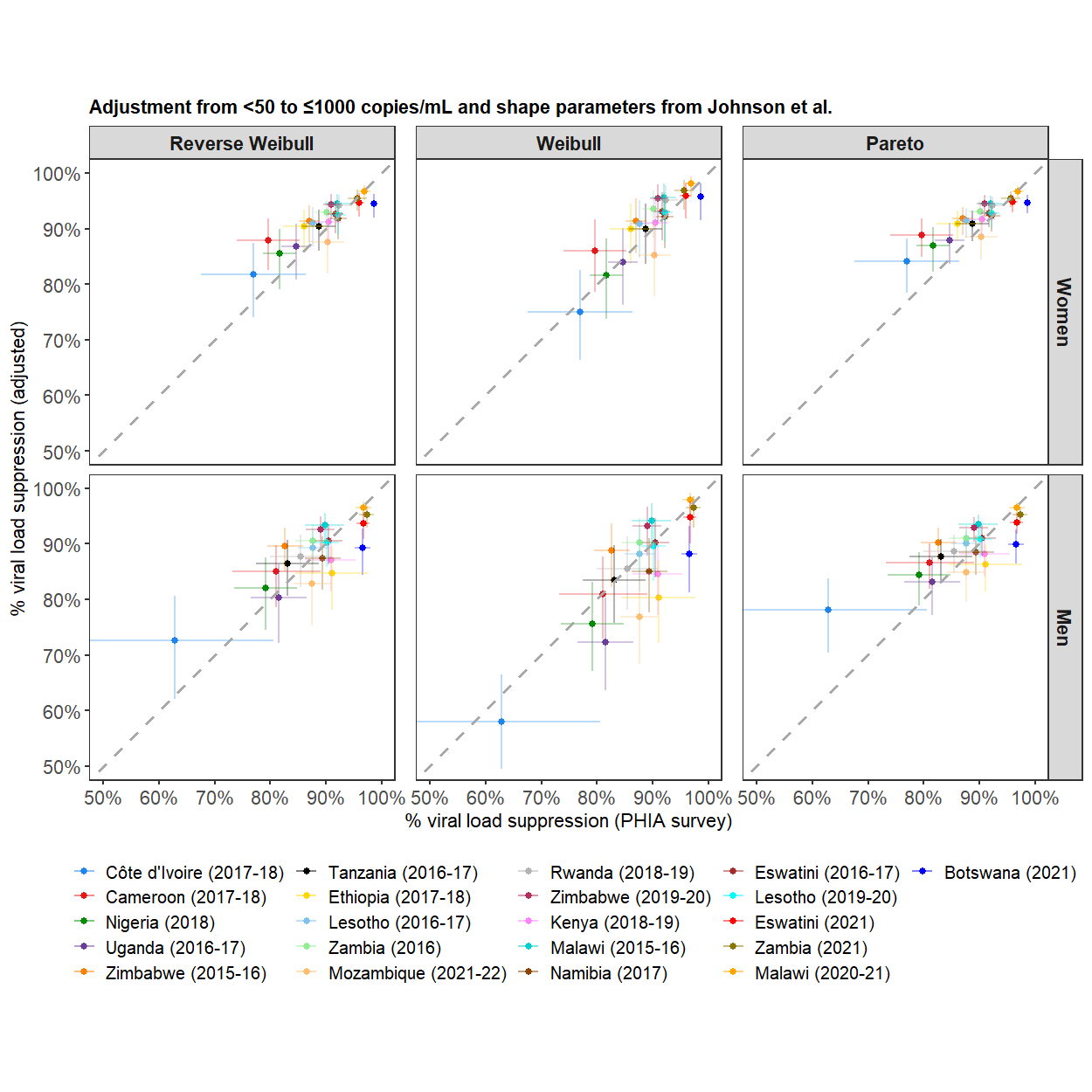


**Figure S7A.** Scatter plots show the relationship between the observed percentage VLS estimates from the individual patient data in the PHIA surveys and the adjusted estimates (from <50 to ≤1000 copies/mL) by sex using the reverse Weibull, Weibull and Pareto models and parameters from Johnson *et al.* Surveys in legend are sequenced in increasing order of observed VLS.


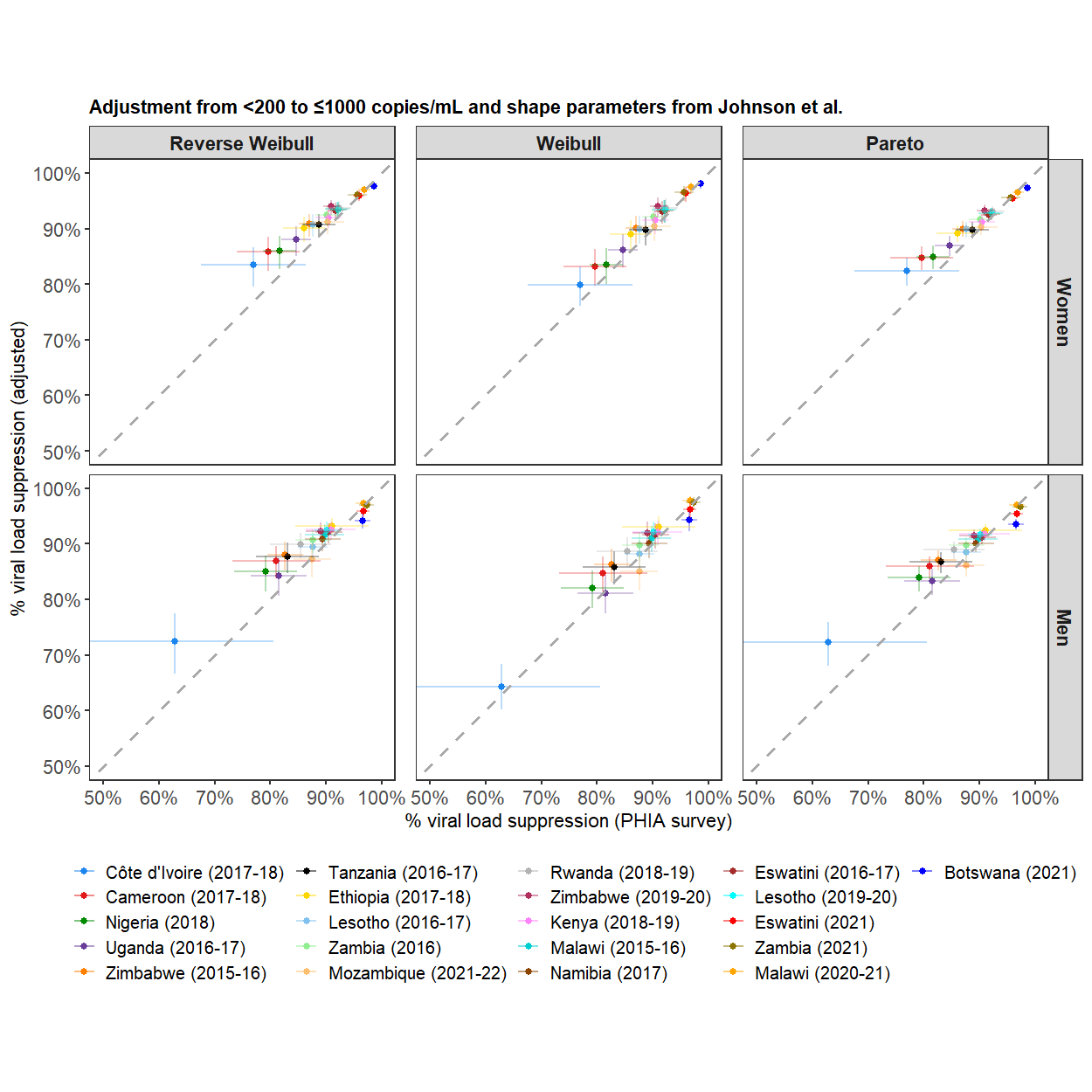


**Figure 7B.** Scatter plots show the relationship between the observed percentage VLS estimates from the individual patient data in the PHIA surveys and the adjusted estimates (from <200 to ≤1000 copies/mL) by sex using the reverse Weibull, Weibull and Pareto models and parameters from Johnson *et al*. Surveys in legend are sequenced in increasing order of observed VLS


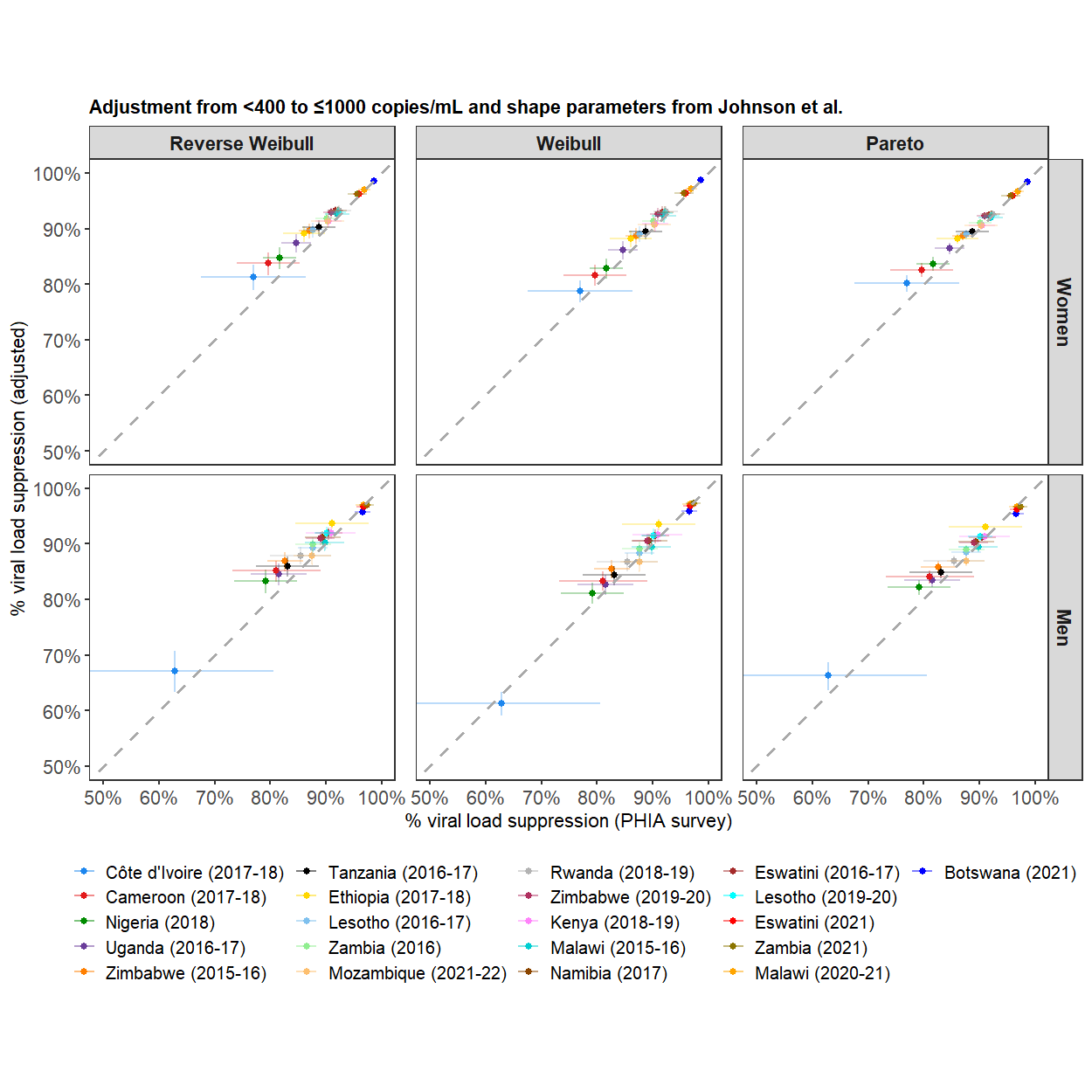


**Figure S7C.** Scatter plots show the relationship between the observed percentage VLS estimates from the individual patient data in the PHIA surveys and the adjusted estimates (from <400 to ≤1000 copies/mL) by sex using the reverse Weibull, Weibull and Pareto models and parameters from Johnson *et al.* Surveys in legend are sequenced in increasing order of observed VLS.


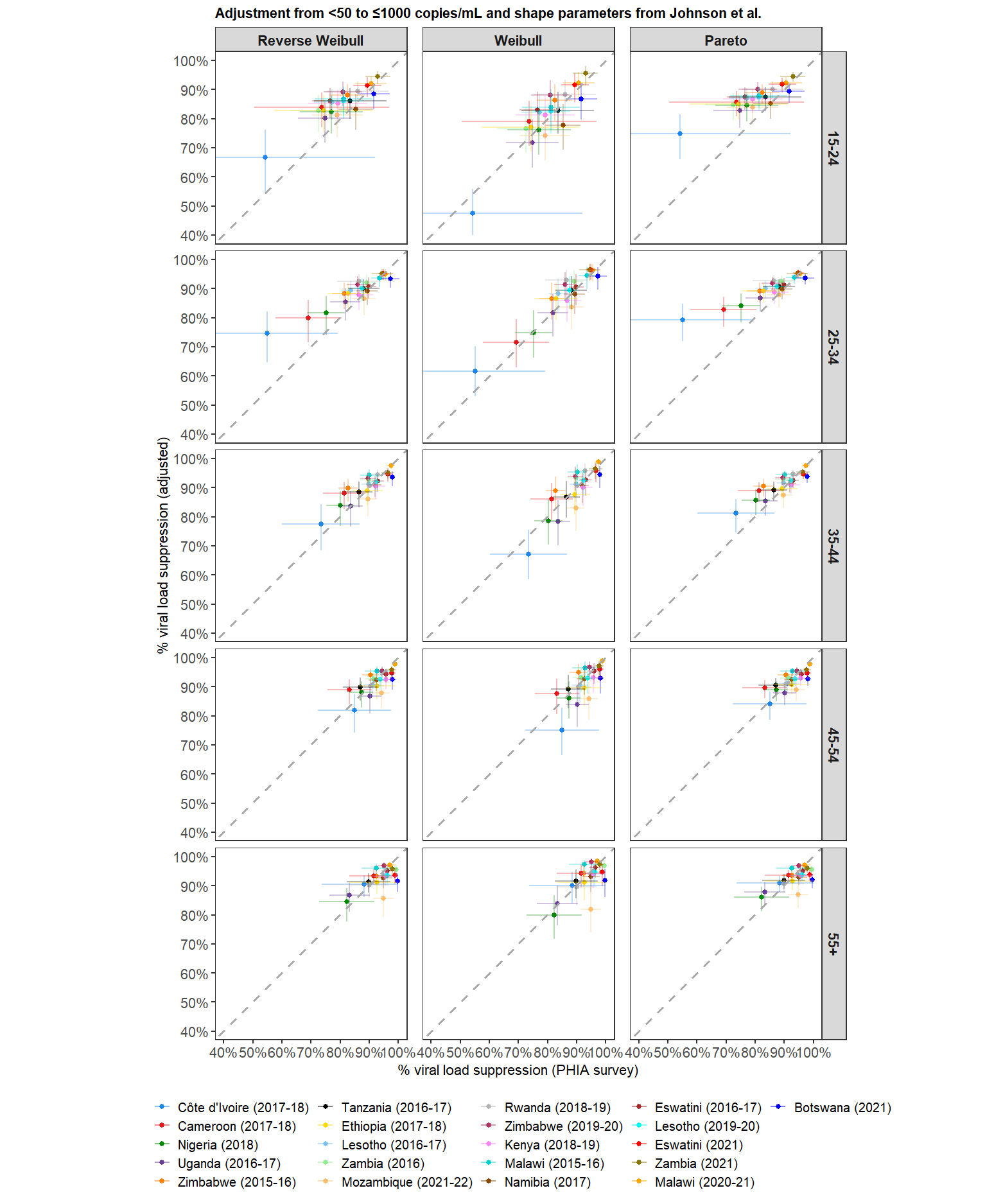


**Figure S8A.** Scatter plots show the relationship between the observed percentage VLS estimates from the individual patient data in the PHIA surveys and the adjusted estimates (from <50 to ≤1000 copies/mL) by age using the reverse Weibull, Weibull and Pareto models and parameters from Johnson *et al.* Surveys in legend are sequenced in increasing order of observed VLS.


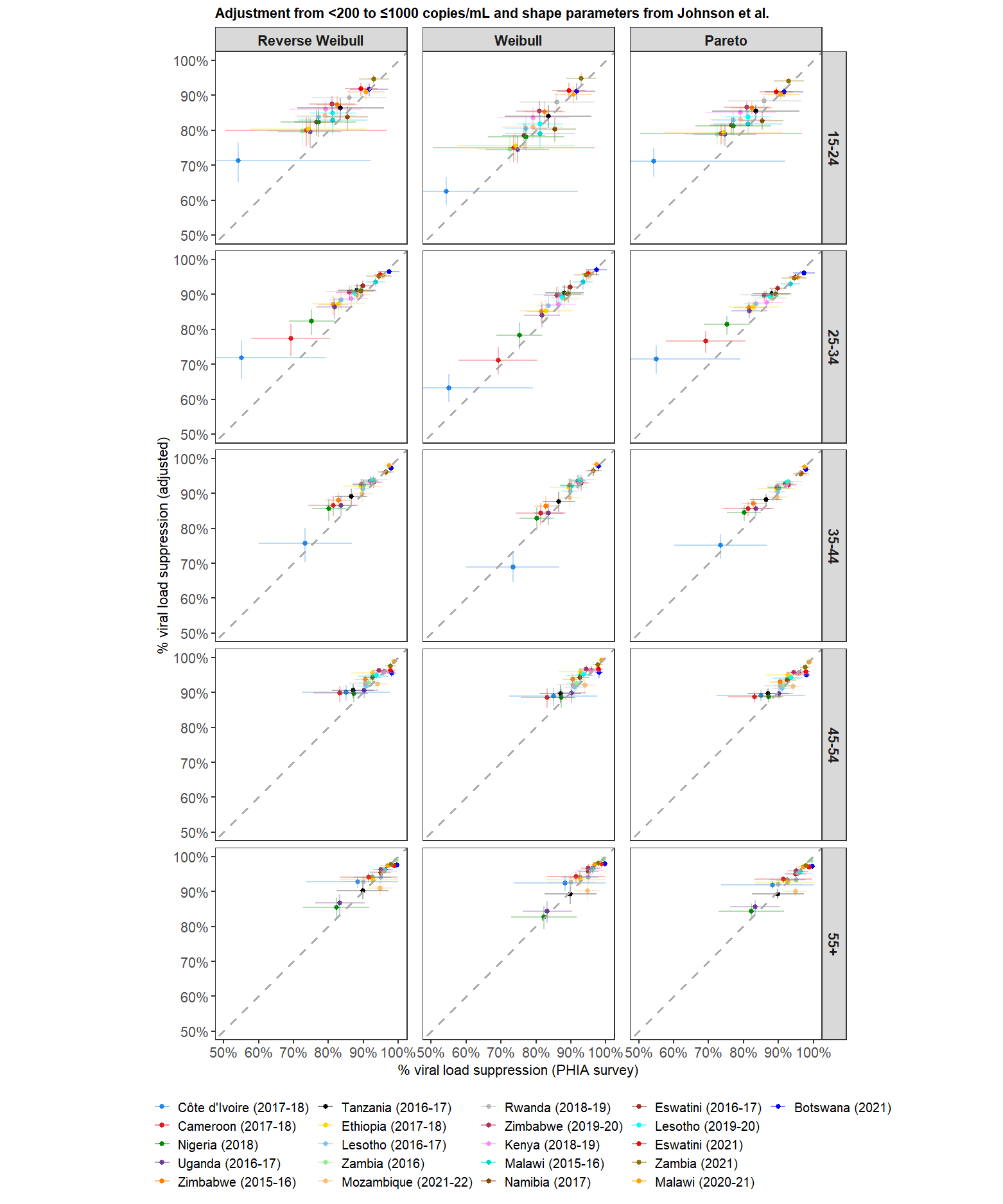


**Figure S8B.** Scatter plots show the relationship between the observed percentage VLS estimates from the individual patient data in the PHIA surveys and the adjusted estimates (from <200 to ≤1000 copies/mL) by age using the reverse Weibull, Weibull and Pareto models and parameters from Johnson *et al.* Surveys in legend are sequenced in increasing order of observed VLS.


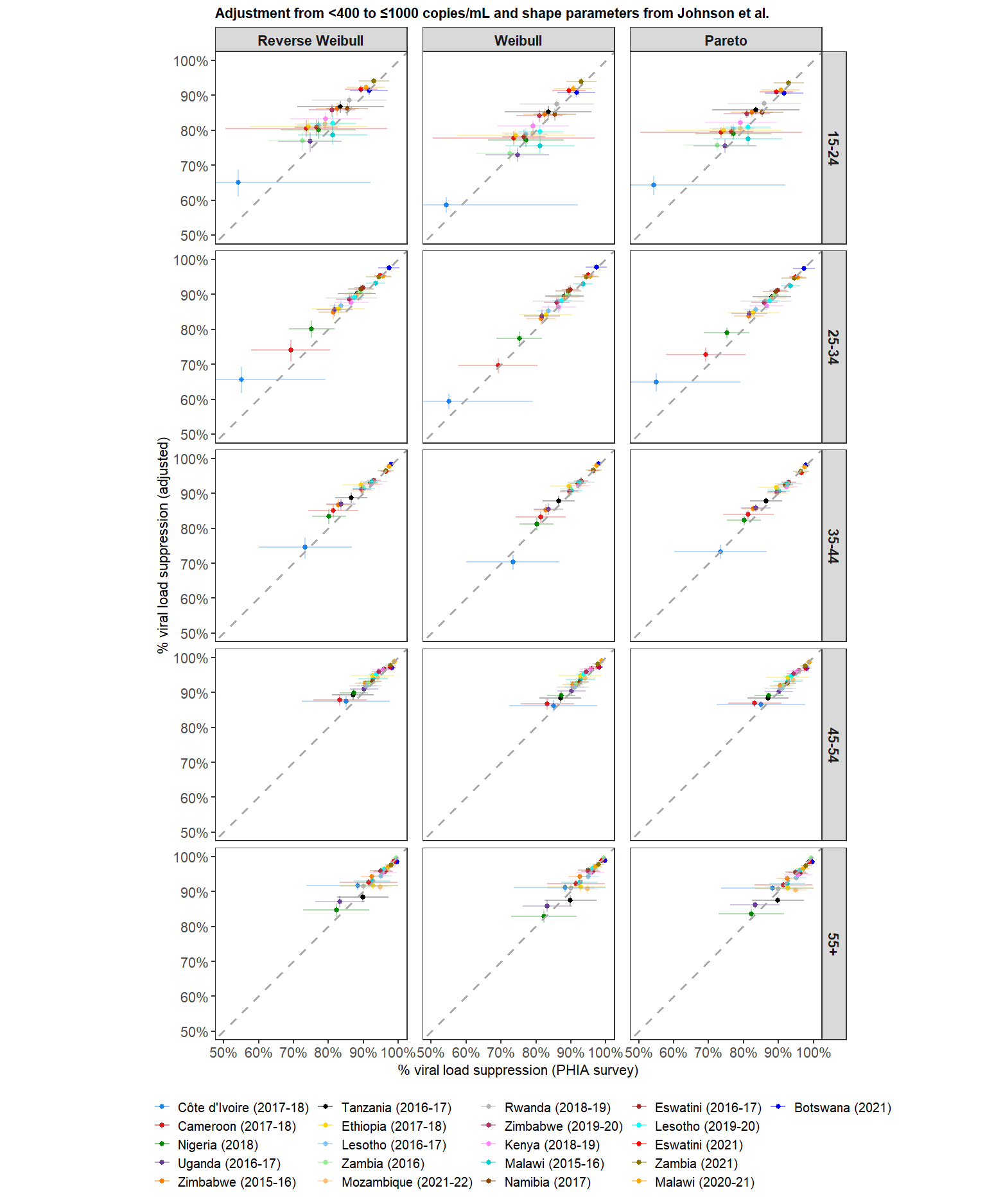


**Figure S8C.** Scatter plots show the relationship between the observed percentage VLS estimates from the individual patient data in the PHIA surveys and the adjusted estimates (from <400 to ≤1000 copies/mL) by age using the reverse Weibull, Weibull and Pareto models and parameters from Johnson *et al.* Surveys in legend are sequenced in increasing order of observed VLS.

**Table S3.** Root-mean-squared error (which quantifies the square root of the variance of residuals between the predicted values from the model and actual values from the PHIA survey data). A lower RMSE indicates better model fit and more precise model predictions. A shape parameter of 1.20 was used for the pooled, sex- and age-stratified comparisons for the Pareto model.

| **Threshold** | **Category** | **Root-mean-squared error (RMSE)** | | | | | |  |  |  |
| --- | --- | --- | --- | --- | --- | --- | --- | --- | --- | --- |
|  |  | **Shape parameters from Johnson *et al.*** | | | **Shape parameters from calibration to PHIA surveys** | | | **Shape parameters from calibration to PHIA surveys** | | |
|  |  | Reverse Weibull | Weibull | Pareto | Reverse Weibull | Weibull | Pareto* | Fréchet | Gamma | Lognormal |
| <50 | Pooled | 3.2% | 3.0% | 3.8% | 3.6% | 4.3% | 3.0% | 3.5% | 3.1% | 3.1% |
| <200 | Pooled | 3.1% | 1.9% | 2.5% | 3.4% | 1.4% | 1.4% | 2.3% | 2.2% | 2.3% |
| <400 | Pooled | 2.1% | 1.1% | 1.4% | 2.3% | 0.8% | 0.9% | 1.3% | 1.3% | 1.3% |
| <50 | Men | 4.2% | 5.2% | 5.0% | 4.2% | 5.2% | 4.9% | 4.5% | 4.4% | 4.1% |
|  | Women | 3.2% | 2.9% | 3.7% | 3.7% | 3.1% | 2.6% | 3.3% | 3.0% | 3.1% |
| <200 | Men | 3.7% | 2.1% | 3.2% | 3.8 % | 2.8% | 2.1% | 3.0% | 2.8% | 2.9% |
|  | Women | 2.9% | 1.9% | 2.3% | 3.3% | 2.0% | 1.3% | 2.1% | 1.9% | 2.1% |
| <400 | Men | 2.3% | 1.3% | 1.7% | 2.4% | 1.7% | 1.1% | 1.6% | 1.7% | 1.6% |
|  | Women | 2.0% | 1.1% | 1.3% | 2.2% | 1.2% | 0.8% | 1.2% | 1.1% | 1.2% |
| <50 | 15-24 | 6.5% | 4.2% | 8.5% | 5.1% | 6.1% | 4.7% | 4.8% | 4.5% | 4.3% |
|  | 25-34 | 6.0% | 3.4% | 7.4% | 5.1% | 4.5% | 4.8% | 5.6% | 3.4% | 4.4% |
|  | 35-44 | 3.2% | 3.6% | 3.7% | 3.2% | 3.7% | 3.3% | 3.4% | 3.6% | 3.2% |
|  | 45-54 | 3.0% | 3.9% | 2.8% | 3.3% | 3.5% | 4.3% | 2.9% | 3.5% | 3.1% |
|  | 55+ | 3.6% | 3.9% | 3.5% | 4.1% | 3.6% | 4.8% | 3.5% | 3.6% | 3.4% |
| <200 | 15-24 | 5.9% | 3.0% | 5.3% | 5.0% | 2.7% | 3.5% | 3.7% | 3.2% | 3.4% |
|  | 25-34 | 5.2% | 2.7% | 4.7% | 4.6% | 3.5% | 3.2% | 3.8% | 2.8% | 3.2% |
|  | 35-44 | 2.6% | 2.0% | 2.0% | 2.7% | 2.4% | 1.3% | 2.0% | 2.2% | 2.0% |
|  | 45-54 | 2.5% | 2.2% | 2.1% | 3.5% | 2.7% | 1.9% | 2.4% | 2.7% | 2.6% |
|  | 55+ | 2.0% | 1.8% | 1.8% | 3.2% | 2.1% | 1.9% | 2.1% | 2.1% | 2.0% |
| <400 | 15-24 | 4.2% | 2.5% | 3.5% | 3.7% | 2.4% | 2.7% | 2.7% | 2.6% | 2.6% |
|  | 25-34 | 3.4% | 1.5% | 2.7% | 2.9% | 1.9% | 1.8% | 2.1% | 1.5% | 1.8% |
|  | 35-44 | 1.9% | 1.3% | 1.3% | 1.9% | 1.5% | 1.0% | 1.3% | 1.4% | 1.3% |
|  | 45-54 | 1.7% | 1.3% | 1.2% | 2.4% | 1.6% | 1.0% | 1.4% | 1.6% | 1.5% |
|  | 55+ | 1.7% | 1.6% | 1.6% | 2.4% | 1.6% | 1.8% | 1.7% | 1.6% | 1.6% |

*Shape parameter of 1.20 from visual calibration to PHIA survey used for pooled, sex- and age-stratified predictions.

**Table S4.** Average bias (which quantifies the average difference between the models’ predicted VLS point estimate, and the actual survey point estimate). Note: negative values indicate underestimation while positive values indicate overestimation.

| Threshold | Category | **Average bias** | | | | | |  |  |  |
| --- | --- | --- | --- | --- | --- | --- | --- | --- | --- | --- |
|  |  | **Johnson *et al.*** | | | **Calibration to PHIA surveys** | | | **Calibration to PHIA surveys** | | |
|  |  | Reverse Weibull | Weibull | Pareto | Reverse Weibull | Weibull | Pareto | Fréchet | Gamma | Lognormal |
| <50 | Pooled | 1.3% | 0.4% | 2.0% | 1.9% | -3.4% | 1.2% | 1.7% | 1.0% | 1.6% |
| <200 | Pooled | 2.3% | 1.5% | 1.5% | 2.6% | -0.8% | 0.1% | 1.5 % | 1.8% | 1.7% |
| <400 | Pooled | 1.6% | 0.8% | 0.8% | 1.8% | -0.4% | 0.2% | 0.8% | 1.0% | 0.9% |
| <50 | Men | 0.5% | -2.1% | 1.6% | 0.7% | 0.2% | -2.2% | 1.4% | 0.2% | 0.9% |
|  | Women | 1.7% | 1.3% | 2.2% | 2.3% | 1.6% | -0.7% | 1.9% | 1.4% | 1.9% |
| <200 | Men | 2.5% | 1.2% | 1.8% | 2.6% | 2.2% | 0.1% | 1.8% | 2.2% | 2.1% |
|  | Women | 2.2% | 1.5% | 1.5% | 2.6% | 1.7% | 0.1% | 1.4% | 1.6% | 1.6% |
| <400 | Men | 1.7% | 0.7% | 0.9% | 1.8% | 1.2% | 0.05% | 1.0% | 1.2% | 1.1% |
|  | Women | 1.8% | 0.7% | 0.9% | 1.7% | 1.0% | <0.0001% | 0.7% | 0.9% | 0.8% |
| <50 | 15-24 | 5.2% | 1.0% | 6.8% | 3.4% | -4.3% | 2.3% | 3.1% | 1.8% | 2.5% |
|  | 25-34 | 3.5% | 1.8% | 4.5% | 2.4% | 3.4% | 1.0% | 3.0% | 1.8% | 2.6% |
|  | 35-44 | 1.0% | 0.03% | 1.7% | 1.1% | 1.5% | -1.4% | 1.5% | 0.6% | 1.3% |
|  | 45-54 | -0.8% | -1.0% | -0.3% | 1.6% | 0.4% | -3.1% | 0.6% | 0.3% | 0.7% |
|  | 55+ | -1.1% | -1.0% | -0.7% | 2.0% | 0.5% | -3.2% | 0.7% | 0.5% | 0.7% |
| <200 | 15-24 | 4.6% | 1.5% | 3.7% | 3.6% | -0.9% | 1.3% | 1.9% | 1.9% | 1.9% |
|  | 25-34 | 3.7% | 2.1% | 2.9% | 3.1% | 2.9% | 1.1% | 2.2% | 2.1% | 2.2% |
|  | 35-44 | 1.9% | 1.0% | 1.2% | 2.0% | 1.7% | -0.2% | 1.2% | 1.3% | 1.3% |
|  | 45-54 | 1.4% | 1.2% | 0.7% | 2.5% | 1.8% | -0.3% | 1.3% | 1.8% | 1.6% |
|  | 55+ | 0.6% | 0.5% | 0.04% | 2.2% | 1.1% | -0.9% | 0.7% | 1.1% | 0.9% |
| <400 | 15-24 | 3.1% | 0.9% | 2.1% | 2.5% | -0.4% | 0.7% | 1.0% | 1.1% | 1.1% |
|  | 25-34 | 2.4% | 1.1% | 1.5% | 2.0% | 1.5% | 0.5% | 1.1% | 1.1% | 1.1% |
|  | 35-44 | 1.4% | 0.6% | 0.6% | 1.4% | 1.0% | -0.1% | 0.6% | 0.8% | 0.7% |
|  | 45-54 | 1.1% | 0.8% | 0.5% | 1.8% | 1.1% | -0.08% | 0.8% | 1.1% | 1.0% |
|  | 55+ | 0.3% | 0.02% | -0.3% | 1.3% | 0.4% | -0.8% | 0.1% | 0.4% | 0.3% |

**Table S5.** Comparing root-mean-squared error in adjustments from <50, <200 and <400 to ≤1000 copies/mL by sex and age using the Fréchet, gamma and lognormal models and common versus sex- and age-specific shape parameters from calibration to the PHIA survey data.

| **Threshold** | **Category** | **Root-mean-squared error (RMSE)** | | | | | |
| --- | --- | --- | --- | --- | --- | --- | --- |
|  |  | **Common shape parameter from calibration to PHIA survey data** | | | **Age- and sex-specific shape parameters from calibration to PHIA survey data** | | |
|  |  | Fréchet | Gamma | Lognormal | Fréchet | Gamma | Lognormal |
| <50 | Men | 4.3% | 5.0% | 4.0% | 4.5% | 4.4% | 4.1% |
|  | Women | 3.5% | 3.2% | 3.3% | 3.3% | 3.0% | 3.1% |
| <200 | Men | 2.8% | 2.3% | 2.6% | 3.0% | 2.8% | 2.9% |
|  | Women | 2.2% | 2.1% | 2.2% | 2.1% | 1.9% | 2.1% |
| <400 | Men | 1.5% | 1.5% | 1.5% | 1.6% | 1.7% | 1.6% |
|  | Women | 1.3% | 1.2% | 1.3% | 1.2% | 1.1% | 1.2% |
| <50 | 15-24 | 7.1% | 4.5% | 5.4% | 4.8% | 4.5% | 4.3% |
|  | 25-34 | 6.4% | 3.7% | 5.1% | 5.6% | 3.4% | 4.4% |
|  | 35-44 | 3.4% | 3.6% | 3.2% | 3.4% | 3.6% | 3.2% |
|  | 45-54 | 2.9% | 3.7% | 3.1% | 2.9% | 3.5% | 3.1% |
|  | 55+ | 3.5% | 3.8% | 3.5% | 3.5% | 3.6% | 3.4% |
| <200 | 15-24 | 4.7% | 3.2% | 3.9% | 3.7% | 3.2% | 3.4% |
|  | 25-34 | 4.2% | 3.0% | 3.6% | 3.8% | 2.8% | 3.2% |
|  | 35-44 | 1.9% | 2.2% | 2.0% | 2.0% | 2.2% | 2.0% |
|  | 45-54 | 2.1% | 2.4% | 2.3% | 2.4% | 2.7% | 2.6% |
|  | 55+ | 1.8% | 1.9% | 1.8% | 2.1% | 2.1% | 2.0% |
| <400 | 15-24 | 3.2% | 2.6% | 2.8% | 2.7% | 2.6% | 2.6% |
|  | 25-34 | 2.4% | 1.6% | 2.0% | 2.1% | 1.5% | 1.8% |
|  | 35-44 | 1.3% | 1.4% | 1.3% | 1.3% | 1.4% | 1.3% |
|  | 45-54 | 1.2% | 1.4% | 1.3% | 1.4% | 1.6% | 1.5% |
|  | 55+ | 1.6% | 1.6% | 1.6% | 1.7% | 1.6% | 1.6% |

**Table S6.** Comparing average bias in adjustments from <50, <200 and <400 to ≤1000 copies/mL by sex and age using the Fréchet, gamma and lognormal models and common versus sex- and age-specific shape parameters from calibration to the PHIA survey data.

| **Threshold** | **Category** | **Average bias** | | | | | |
| --- | --- | --- | --- | --- | --- | --- | --- |
|  |  | **Common shape parameter from calibration to PHIA survey data** | | | **Age- and sex-specific shape parameters from calibration to PHIA survey data** | | |
|  |  | Fréchet | Gamma | Lognormal | Fréchet | Gamma | Lognormal |
| <50 | Men | 1.0% | -1.4% | 0.06% | 1.4% | 0.2% | 0.9% |
|  | Women | 2.0% | 1.9% | 2.2% | 1.9% | 1.4% | 1.9% |
| <200 | Men | 1.6% | 1.6% | 1.7% | 1.8% | 2.2% | 2.1% |
|  | Women | 1.5% | 1.8% | 1.7% | 1.4% | 1.6% | 1.6% |
| <400 | Men | 0.9% | 0.9% | 0.9% | 1.0% | 1.2% | 1.1% |
|  | Women | 0.7% | 1.0% | 0.9% | 0.7% | 0.9% | 0.8% |
| <50 | 15-24 | 5.7% | 1.7% | 4.0% | 3.1% | 1.8% | 2.5% |
|  | 25-34 | 4.0% | 2.4% | 3.5% | 3.0% | 1.8% | 2.6% |
|  | 35-44 | 1.4% | 0.07% | 1.3% | 1.5% | 0.6% | 1.3% |
|  | 45-54 | -0.4% | -0.5% | -0.3% | 0.6% | 0.3% | 0.7% |
|  | 55+ | -0.7% | -0.4% | -0.4% | 0.7% | 0.5% | 0.7% |
| <200 | 15-24 | 3.3% | 1.9% | 2.7% | 1.9% | 1.9% | 1.9% |
|  | 25-34 | 2.7% | 2.4% | 2.6% | 2.2% | 2.1% | 2.2% |
|  | 35-44 | 1.1% | 1.3% | 1.3% | 1.2% | 1.3% | 1.3% |
|  | 45-54 | 0.8% | 1.5% | 1.2% | 1.3% | 1.8% | 1.6% |
|  | 55+ | 0.08% | 0.7% | 0.5% | 0.7% | 1.1% | 0.9% |
| <400 | 15-24 | 1.8% | 1.1% | 1.5% | 1.0% | 1.1% | 1.1% |
|  | 25-34 | 1.4% | 1.3% | 1.4% | 1.1% | 1.1% | 1.1% |
|  | 35-44 | 0.6% | 0.8% | 0.7% | 0.6% | 0.8% | 0.7% |
|  | 45-54 | 0.5% | 0.9% | 0.8% | 0.8% | 1.1% | 1.0% |
|  | 55+ | -0.2% | 0.2% | -0.00004% | 0.1% | 0.4% | 0.3% |

**Table S7**. Sensitivity analyses showing shape parameter estimates (95% CI) for Weibull, reverse Weibull, Pareto, Fréchet, Gamma, and Lognormal models fitted to PHIA survey data, comparing inclusion of all PLHIV on ART in 21 PHIAs, PLHIV on ART for ≥12 months in 14 PHIAs (2015–2019), and all PLHIV on ART in 14 PHIAs (2015–2019).

| **Model** | **Johnson *et al.*** | **21 surveys (2015-2022) including all PLHIV on ART** | **14 surveys (2015–2019), including PLHIV on ART ≥12 months** | **14 surveys (2015–2019), including all PLHIV** |
| --- | --- | --- | --- | --- |
| Reverse Weibull | 2.81 (1.70, 3.92) | 2.98 (2.93, 3.02) | 2.41 (2.35, 2.48) | 2.49 (2.45, 2.59) |
| Weibull | 0.85 (0.43, 1.26) | 0.91 (0.90, 0.92) | 0.84 (0.81, 0.86) | 0.90 (0.88, 0.91) |
| Pareto | 1.73 (1.20, 2.26) | - | - | - |
| Fréchet | - | 1.86 (1.83, 1.90) | 1.71 (1.65, 1.76) | 1.72 (1.68, 1.78) |
| Gamma | - | 0.81 (0.79, 0.84) | 0.67 (0.64, 0.70) | 0.78 (0.75, 0.81) |
| Lognormal | - | 0.89 (0.88, 0.90) | 0.89 (0.88, 0.90) | 0.93 (0.91, 0.95) |

**Table S8**. Numbers of included participants by duration on ART, with survey‑weighted estimates of viral load suppression (VLS) among all PLHIV on ART and among those self‑reporting ≥12 months of ART use.

| Survey | PLHIV on all PLHIV on ART | PLHIV on ART ≥12 months, n (%) | PLHIV on ART <12 months, n (%) | ART duration information unavailable, n (%) | Survey VLS among all PLHIV on ART (% ≤1000 copies/mL) (95% CI) | Survey VLS among PLHIV on ART ≥12 months (% ≤1000 copies/mL) (95% CI) |
| --- | --- | --- | --- | --- | --- | --- |
| Cameroon (2017-2018) | 493 | 277 (56.2) | 89 (18.1) | 127 (25.8) | 80.1 (75.1, 85.0) | 82.6 (77.4, 87.7) |
| Côte d'Ivoire (2017-2018) | 207 | 93 (44.9) | 34 (16.4) | 80 (38.6) | 73.7 (64.3, 83.0) | 74.9 (63.7, 86.2) |
| Eswatini (2016-2017) | 2369 | 1845 (77.9) | 381 (16.1) | 143 (6.0) | 91.4 (90.3, 92.5) | 92.8 (91.6, 94.0) |
| Ethiopia (2017-2018) | 476 | 370 (77.7) | 24 (5.0) | 82 (17.2) | 87.6 (84.1, 91.0) | 89.9 (86.5, 93.3) |
| Kenya (2018-2019) | 1176 | 899 (76.4) | 136 (11.6) | 141 (12.0) | 90.6 (88.6, 92.6) | 92.5 (90.4, 94.6) |
| Lesotho (2016-2017) | 2435 | 1653 (67.9) | 530 (21.8) | 252 (10.3) | 87.7 (86.1, 89.3) | 89.4 (87.7, 91.0) |
| Malawi (2015-2016) | 1564 | 1198 (76.6) | 218 (13.9) | 148 (9.5) | 91.3 (89.3, 93.3) | 91.4 (89.4, 93.5) |
| Namibia (2017) | 2069 | 1469 (71.0) | 232 (11.2) | 368 (17.8) | 91.3 (89.8, 92.9) | 91.5 (89.7, 93.3) |
| Nigeria (2018) | 1322 | 577 (43.6) | 125 (9.5) | 620 (46.9) | 80.9 (78.2, 83.6) | 83.1 (79.0, 87.2) |
| Rwanda (2018-2019) | 767 | 598 (78.0) | 51 (6.6) | 118 (15.4) | 90.1 (87.6, 92.6) | 90.3 (87.4, 93.3) |
| Tanzania (2016-2017) | 1022 | 641 (62.7) | 112 (11.0) | 269 (26.3) | 87.2 (84.5, 89.8) | 87.6 (84.4, 90.8) |
| Uganda (2016-2017) | 1185 | 831 (70.1) | 137 (11.6) | 217 (18.3) | 83.7 (81.4, 85.9) | 84.8 (82.3, 87.3) |
| Zambia (2016) | 1556 | 1103 (70.9) | 249 (16.0) | 204 (13.1) | 89.2 (87.4, 91.1) | 90.3 (88.2, 92.4) |
| Zimbabwe (2015-2016) | 2497 | 1972 (79.0) | 288 (11.5) | 237 (9.5) | 85.4 (83.6, 87.2) | 87.1 (85.3, 89.0) |

**Figure S9A.** Empirical cumulative distribution functions (CDFs) of observed viral loads among PLHIV on ART (black solid lines) and modelled CDFs using shape parameter estimates from Johnson *et al.* (coloured solid lines) and PHIA calibration (dashed coloured lines), and scale parameters set so that the cumulative probability of a viral load ≤1000 copies/mL matches the VLS estimated from each survey. Results are shown for the A. Côte d'Ivoire (2017–18); B. Nigeria (2018); C. Lesotho (2016–2017); D. Namibia (2018); E. Eswatini (2021); and F. Botswana (2021) PHIA surveys. The x-axis was truncated at 50 copies/mL for visualization (see panel B for full x-axis range). The irregularity of the Côte d’Ivoire curve reflects sparse data due to the small number of PLHIV on ART (n = 207).


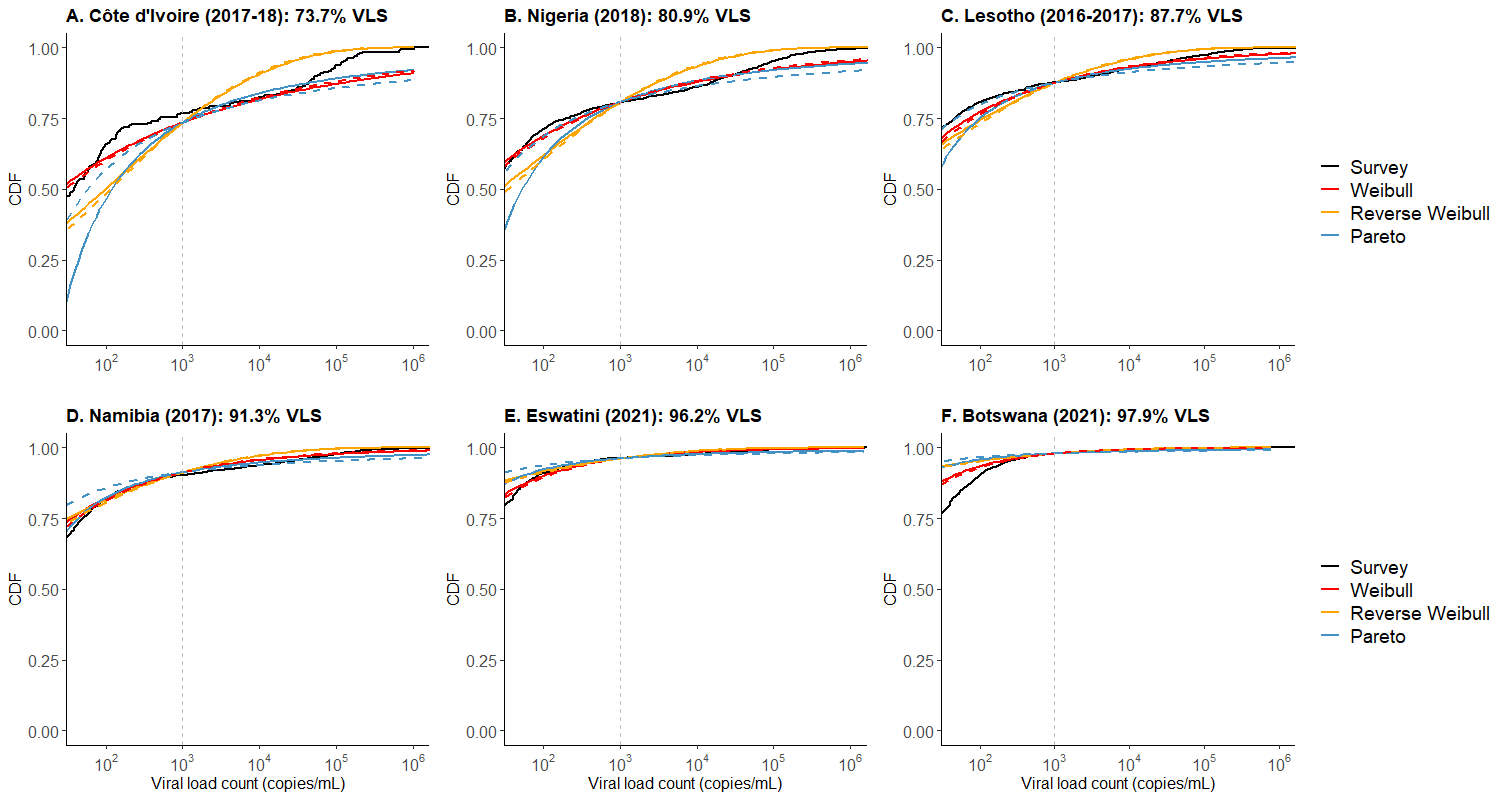


**Figure S9B.** Empirical cumulative distribution functions (CDFs) of observed viral loads among PLHIV on ART (black solid lines) and modelled CDFs using shape parameter estimates from Johnson *et al.* (coloured solid lines) and PHIA calibration (dashed coloured lines), and scale parameters set so that the cumulative probability of a viral load ≤1000 copies/mL matches the VLS estimated from each survey. Results are shown for the A. Côte d'Ivoire (2017–18); B. Nigeria (2018); C. Lesotho (2016–2017); D. Namibia (2018); E. Eswatini (2021); and F. Botswana (2021) PHIA surveys. The irregularity of the Côte d’Ivoire curve reflects sparse data due to the small number of PLHIV on ART (n = 207). Note: because the lower limit of detection in the plotted surveys was 20 copies/mL, CDF values for VL <20 are zero.


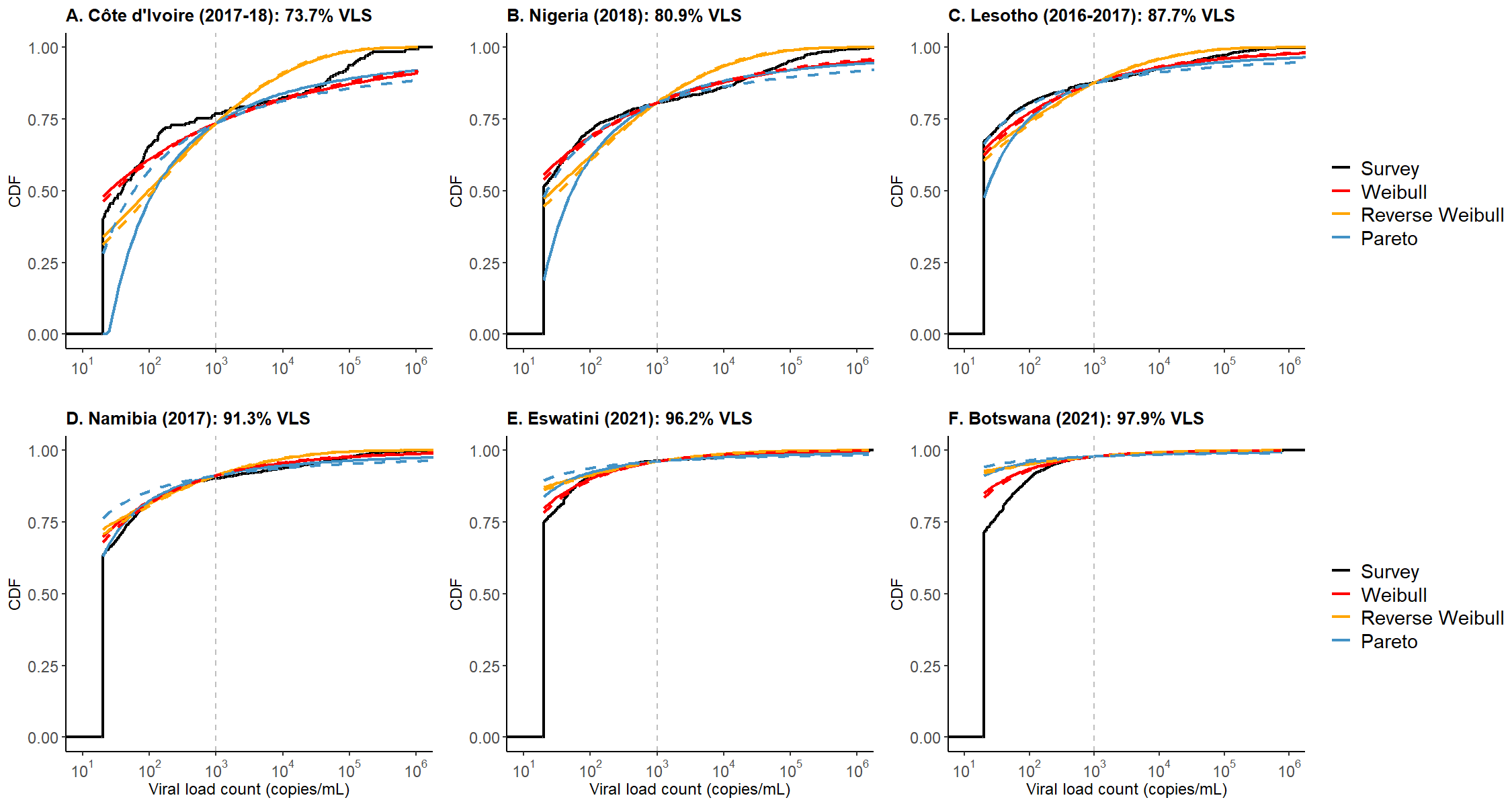


**Figure S10.** Empirical probability distribution functions (PDFs) of observed viral loads among PLHIV on ART (black solid lines) and modelled PDFs using shape parameter estimates from Johnson *et al.* (coloured solid lines) and PHIA calibration (coloured dashed lines), and scale parameters set so that the cumulative probability of a viral load ≤1000 copies/mL matches the VLS estimated from each survey. The lowest observed viral load in the empirical data is 20 copies/mL; therefore, the lines starts at this value. Results are shown for the A. Côte d'Ivoire (2017–18); B. Nigeria (2018); C. Lesotho (2016–2017); D. Namibia (2018); E. Eswatini (2021); and F. Botswana (2021) PHIA surveys. Breaks in the y‑axis were included to allow clearer visualisation of the distribution.

**
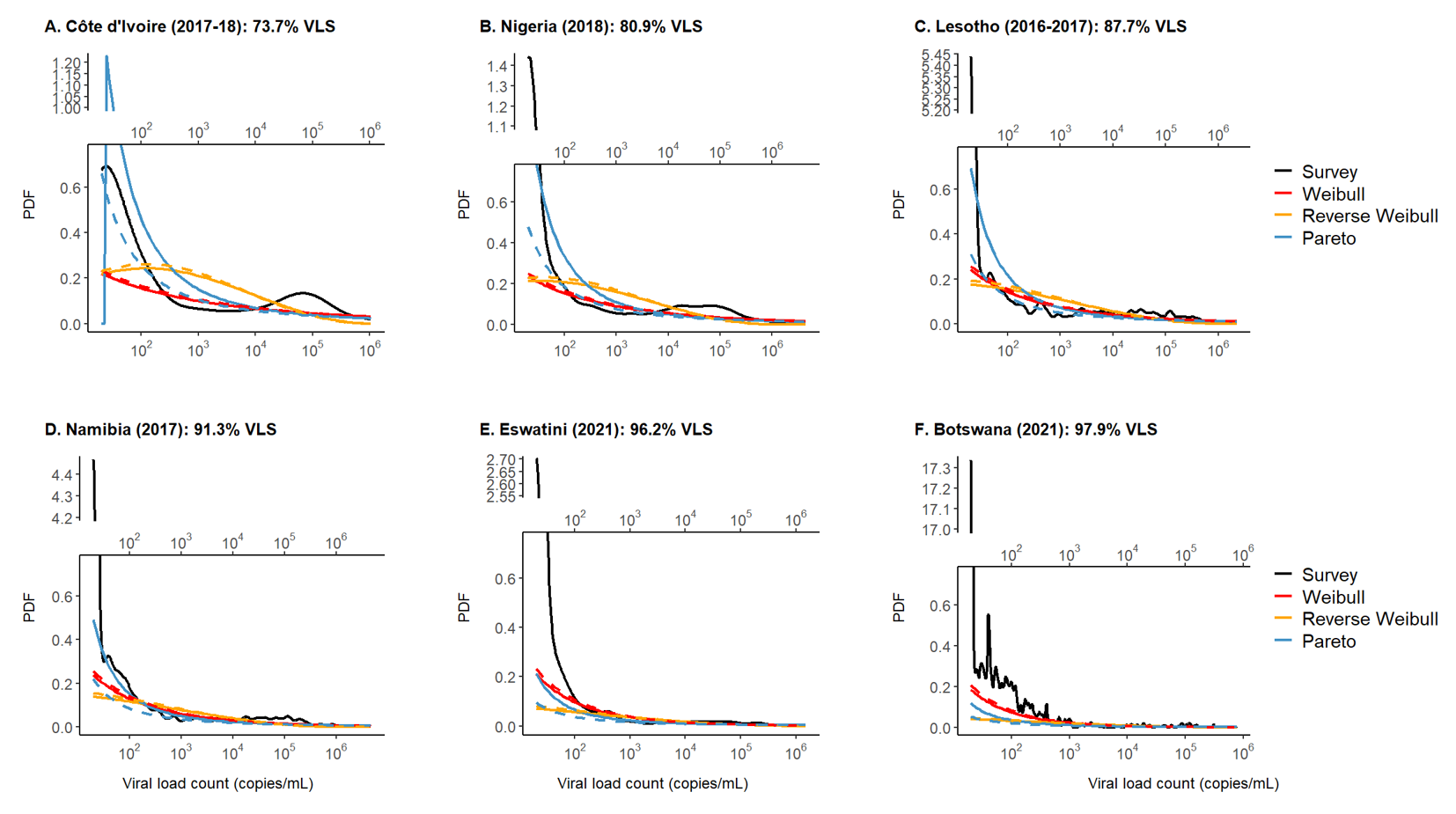
**

**Figure S11.** Histograms show the distribution of observed viral loads among PLHIV on ART in the A. Côte d'Ivoire (2017-18); B. Côte d'Ivoire (2017-18); B. Nigeria (2018); C. Lesotho (2016-2017); D. Namibia (2018); E. Eswatini (2021) and F. Botswana (2021) PHIA surveys. Lines show the probability density estimates for the Pareto, reverse Weibull and Weibull models using shape parameters from Johnson et al. (solid line) and calibration to PHIA surveys (dashed lines). The dashed line for the Pareto model is for shape = 1.20 (see Figure S2 for other values). Note: the scale parameters were set so the cumulative probability of a viral load ≤1000 copies/mL is the same as VLS estimated from the survey data. Gaps in the histogram for Côte d’Ivoire reflect sparse data points for certain viral load values on the x-axis due to the small number of PLHIV on ART (n = 207).


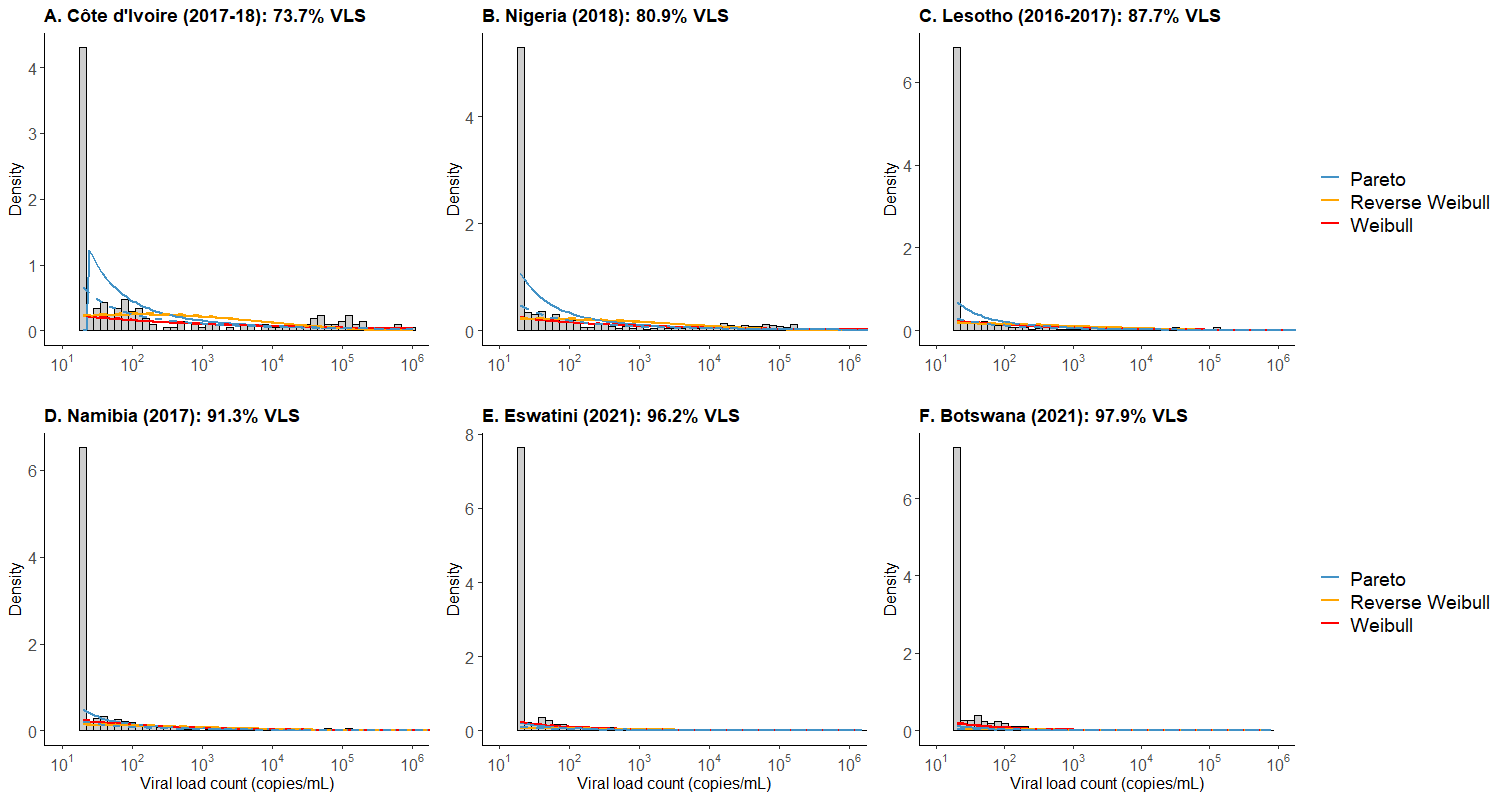

Supplement: Supplementary file 1 [file qai-101-827-s001.docx]
